# Major Population Expansion of East Asians Began before Neolithic Time: Evidence of mtDNA Genomes

**DOI:** 10.1371/journal.pone.0025835

**Published:** 2011-10-06

**Authors:** Hong-Xiang Zheng, Shi Yan, Zhen-Dong Qin, Yi Wang, Jing-Ze Tan, Hui Li, Li Jin

**Affiliations:** 1 State Key Laboratory of Genetic Engineering and MOE Key Laboratory of Contemporary Anthropology, School of Life Sciences and Institutes of Biomedical Sciences, Fudan University, Shanghai, China; 2 Chinese Academy of Sciences and Max-Planck Society (CAS-MPG) Partner Institute for Computational Biology, Shanghai Institutes for Biological Sciences, Chinese Academy of Sciences, Shanghai, China; 3 Key Laboratory of Computational Biology, CAS-MPG Partner Institute for Computational Biology, Chinese Academy of Sciences, Shanghai, China; 4 Human Genome Sequencing Center, Baylor College of Medicine, Houston, Texas, United States of America; University of Cambridge, United Kingdom

## Abstract

It is a major question in archaeology and anthropology whether human populations started to grow primarily after the advent of agriculture, i.e., the Neolithic time, especially in East Asia, which was one of the centers of ancient agricultural civilization. To answer this question requires an accurate estimation of the time of lineage expansion as well as that of population expansion in a population sample without ascertainment bias. In this study, we analyzed all available mtDNA genomes of East Asians ascertained by random sampling, a total of 367 complete mtDNA sequences generated by the 1000 Genome Project, including 249 Chinese (CHB, CHD, and CHS) and 118 Japanese (JPT). We found that major mtDNA lineages underwent expansions, all of which, except for two JPT-specific lineages, including D4, D4b2b, D4a, D4j, D5a2a, A, N9a, F1a1'4, F2, B4, B4a, G2a1 and M7b1'2'4, occurred before 10 kya, i.e., before the Neolithic time (symbolized by Dadiwan Culture at 7.9 kya) in East Asia. Consistent to this observation, the further analysis showed that the population expansion in East Asia started at 13 kya and lasted until 4 kya. The results suggest that the population growth in East Asia constituted a need for the introduction of agriculture and might be one of the driving forces that led to the further development of agriculture.

## Introduction

The invention of agriculture was believed to be critical to the expansion of human populations [1], [2]. East Asia is one of the most important sites for origin of agriculture. The onset of the Neolithic transition in China remains controversial among the archaeologists although the earliest appearance of domesticated crops was found in Dadiwan culture at 7.9 kya (thousands of years ago) when broomcorn millet and foxtail millet were cultivated [3], [4]. Although stronger presence of human activities after Neolithic age suggested recent population expansion after the introduction of agriculture, archeological evidence clearly demonstrated presence of human beings in East Asia throughout the upper Paleolithic period since 40–30 kya [5]. During this period, the last glacial maximum (LGM) occurred at ∼20 kya [6]. At the LGM, unlike the northern and middle part of Europe or North America which were mostly covered with ice sheet or steppe-tundra, most part of China was free of large area of ice sheet [7], indicating that China provided suitable environments for subsistence of ancient human. Upper Paleolithic cultures both before and after the LGM were discovered in China, including Zhoukoudian Upper Cave/Shandingdong (Beijing, 40 kya), Shiyu (Shanxi, 28.9 kya), Xiaonanhai (Henan, 24.1–18.9 kya), Baiyanjiaodong Cave (Guangxi, 14.6–12.1 kya), Maomaodong (Guizhou, 14.6 kya), Xueguan (Shanxi 13.6 kya), etc. [8] In Japan, the incipient Jōmon culture started at ∼14 kya [9], [10].

Despite the abundant upper Paleolithic sites discovered in China, it is not yet clear when the main population expansion occurred. There have been studies on population expansion time in East Eurasia from mitochondrial DNA (mtDNA) [11], [12], and Y chromosomal short tandem repeats (STRs) [13], [14]. Although the time estimations using mtDNA hypervariable region (HVR) are fairly abundant, the HVR contains not enough mutation sites and the mutation sites have too high recurrent rate, which compromised the accuracy of time estimation, especially for those relatively ancient lineages. There were also investigations that focus on one or a few haplogroups of mtDNA, which obtained several complete mtDNA sequences and generated much more precise divergence times between the clades [11], [12], however, due to the picking-up strategy for full sequencing, the expansion time and process in a population could not be easily estimated and need further exploration.

The first trial for unbiased sequencing of large sample of complete mtDNA sequences on a certain population was only available recently [15], in which the authors randomly tested 109 whole sequences from 3 populations in the Philippines. Similar work was done on 205 populations from central Africa including Pygmy tribes [16]. Other studies that sequenced coding region of mtDNA have either sequenced samples from one particular haplogroup of interest to investigate the phylogeny of that haplogroup, or selected some samples with ambiguous haplogroup assignment to determine the definite haplogroup. Few other investigations for natural selection on mtDNA or diversity on a certain population did select random samples, but the sample size was rather small for the relatively high cost of Sanger sequencing method [17], [18].

The 1000 Genome Project [19] provided a wonderful chance for investigating a large number of whole sequences of human mtDNA. Most of the samples were sequenced using the “low coverage sequencing” strategy. Though the average coverage of the autosomes was only 2–6× for the whole genome, the coverage of mtDNA reached hundreds of times due to very high copy number of mitochondria in a single cell. We chose 4 populations from East Asia in this study: Southern Han Chinese (CHS) from Hunan and Fujian Provinces, Beijing Han Chinese (CHB) from Beijing Normal University, Japanese (JPT) from Tokyo metropolitan area, and Denver Han Chinese (CHD) from Denver, Colorado [19], [20], [21], [22]. The former three are Han Chinese, the largest ethnic group in the world and represent the East Asian populations well, and JPT was also considered for the purpose of comparison.

## Results

**Figure 1:**
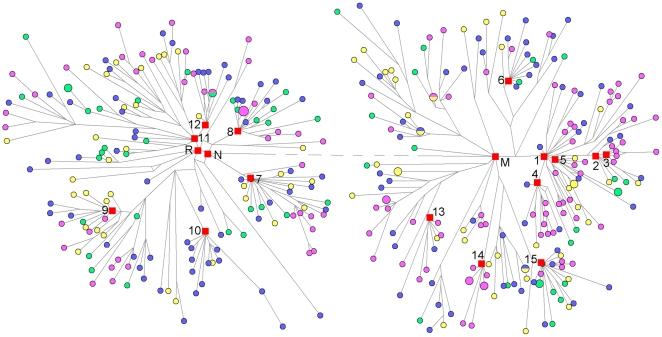
Median-joining network of 367 mtDNA coding region sequences. Median-joining network of 367 mtDNA coding region sequences corresponding to rCRS positions 577-16023. Blue, CHB; green, CHS; yellow, CHD; purple, JPT. Red squares indicate clusters with distinct expansions and Macrohaplogroup M, N and R. 1, D4; 2, D4b2b; 3, D4b2b1; 4, D4a; 5, D4j; 6, D5a2a; 7, A; 8, N9a; 9, F1a1'4; 10, F2; 11, B4; 12, B4a; 13, G2a1; 14, M7a1a; 15, M7b1'2'4. Branch length is proportional to steps of mutations except the dashed line between Macrohaplogroup M and N.

We first estimated the time of lineage expansion using 367 full-genome mtDNA sequences of four available East Asian populations: CHB, CHD, CHS and JPT. After removing the HVS sequences, a median-joining network of mtDNA genomes using Network v4.6 [23] revealed the haplogroup assignments that are consistent with those obtained from Phylotree (see Figure 1 and Methods for details). Macrohaplogroups M and N consist of 53.7% and 46.3% of total samples, respectively (Table 1). The frequencies of haplotypes in the four populations showed no distinct difference with previous work [24], [25]. Star-like clusters (with at least 5 distinct branches splitting from a single node) are common in the network, and most of such clusters consist of individuals from different populations except for two JPT specific clusters (D4b2b1 and M7a1a), suggesting extensive population expansion events in Mainland of East Asia. The time estimated by all three different methods (two ρ statistic-based methods and one Bayesian analysis, see Table 2) showed that except for JPT-specific clusters (D4b2b1 and M7a1a) expanding ∼6 kya, all other star-like clusters (D4, D4b2b, D4a, D4j, D5a2a, A, N9a, F1a1'4, F2, B4, B4a, G2a1, M7b1'2'4) coalesced before 10 kya, which predated the Neolithic time. In particular, D4 and B4 expanded before the LGM. This observation is consistent with earlier estimations on a few mtDNA lineages (A11, M9a'b, F1c) [11], [12] and Y chromosome lineages [10], [14], [26].

**Table 1:** Haplogroup frequencies in the four East Asian populations.

Haplogroup	CHB	CHD	CHS	JPT	Total
	n = 121	n = 73	n = 55	n = 118	n = 367
B4	11.6	16.4	9.1	9.3	11.4
B5	2.5	1.4	10.9	4.2	4.1
N9	5.8	1.4	12.7	8.5	6.8
A	7.4	9.6	7.3	6.8	7.6
R9	1.7	0.0	3.6	0.0	1.1
F	17.4	15.1	9.1	7.6	12.5
R11	1.7	1.4	5.5	0.0	1.6
U	0.8	1.4	0.0	0.0	0.5
HV	0.8	0.0	0.0	0.0	0.3
N10	0.8	0.0	0.0	0.0	0.3
D4	12.4	13.7	10.9	33.1	19.1
D5	9.1	4.1	3.6	2.5	5.2
D6	0.0	0.0	1.8	0.0	0.3
M7	9.9	12.3	12.7	10.2	10.9
C	3.3	6.8	1.8	0.0	2.7
Z	1.7	1.4	3.6	3.4	2.5
M8	2.5	2.7	1.8	1.7	2.2
M9	0.8	2.7	0.0	2.5	1.6
M10	3.3	1.4	1.8	0.0	1.6
M11	0.8	1.4	0.0	0.0	0.5
M12	0.0	1.4	1.8	0.0	0.5
G	5.8	5.5	0.0	10.2	6.3
M33	0.0	0.0	1.8	0.0	0.3

**Table 2:** Age estimations of each cluster with distinct expansion in East Eurasians.

Haplogroup	n	ρ method with Soares complete genome rate^a^	ρ method with Soares synonymous rate^b^	Bayesian MCMC by BEAST^c^
		T(kya)± σ(ky)	T(kya)± σ(ky)	T(95%CI)(ky)
D4	70	26.66±2.78	31.3±5.54	26.86 (19.34, 34.82)
D4b2b	14	12.37±2.34	14.61±5.21	14.2 (10.39, 18.49)
D4b2b1^d^	7	7.50±2.00	6.74±3.18	9.72 (5.9, 13.96)
D4a	12	14.06±3.19	15.08±5.68	14.99 (10.65, 19.66)
D4j	9	17.42±3.28	16.61±5.17	14.46 (9.61, 20.09)
D5a2a	10	14.76±3.51	11.8±6.24	13.4 (9.4, 17.75)
A	28	19.72±2.69	21.92±5.81	20.55 (13.24, 28.64)
N9a	21	16.84±3.05	17.61±5.34	16.54 (11.46, 21.94)
F1a1'4	11	17.71±4.21	15.02±5.30	12.97 (9.35, 16.8)
F2	12	20.92±3.16	22.30±5.71	15.78 (10.86, 21.38)
B4	42	36.24±3.23	40.10±7.27	37.57 (26.61, 48.94)
B4a	10	20.15±4.21	18.89±5.78	15.65 (10.8, 21.62)
G2a1	7	16.97±2.64	20.24±5.50	13.16 (8.93, 17.39)
M7a1a^d^	8	7.21±2.56	9.84±5.73	12.46 (7.75, 17.53)
M7b1'2'4	20	14.62±2.88	11.41±3.99	15.01 (11.02, 19.36)

a Coalescence time were estimated based on ρ statistics with Soares complete genome rate,

b on ρ statistics with Soares synonymous rate,

c using Bayesian MCMC method by BEAST.

*n* indicates number of sequence.

d indicates JPT-specific lineages.

**Figure 2:**
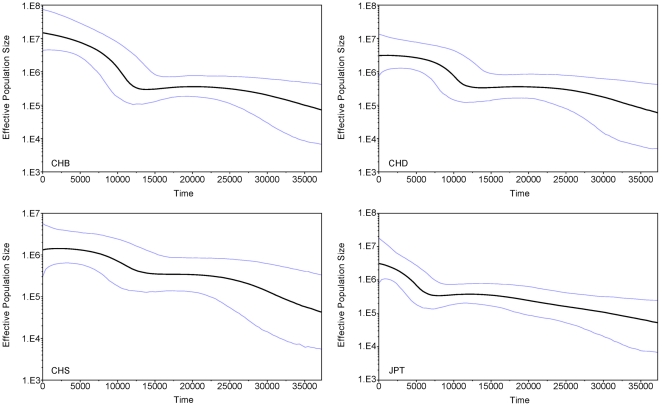
mtDNA Bayesian skyline plot showing the size trend of 4 East Eurasian populations. The y-axis is the product of maternal effective size and generation time. The x-axis is the time from present in units of years. The thick solid line is the median estimate and the thin lines (blue) show the 95% highest posterior density limits estimated with 40,000,000 chains with the first 4,000,000 generations regarded as burn-in. Detailed settings refers to Methods.

**Figure 3:**
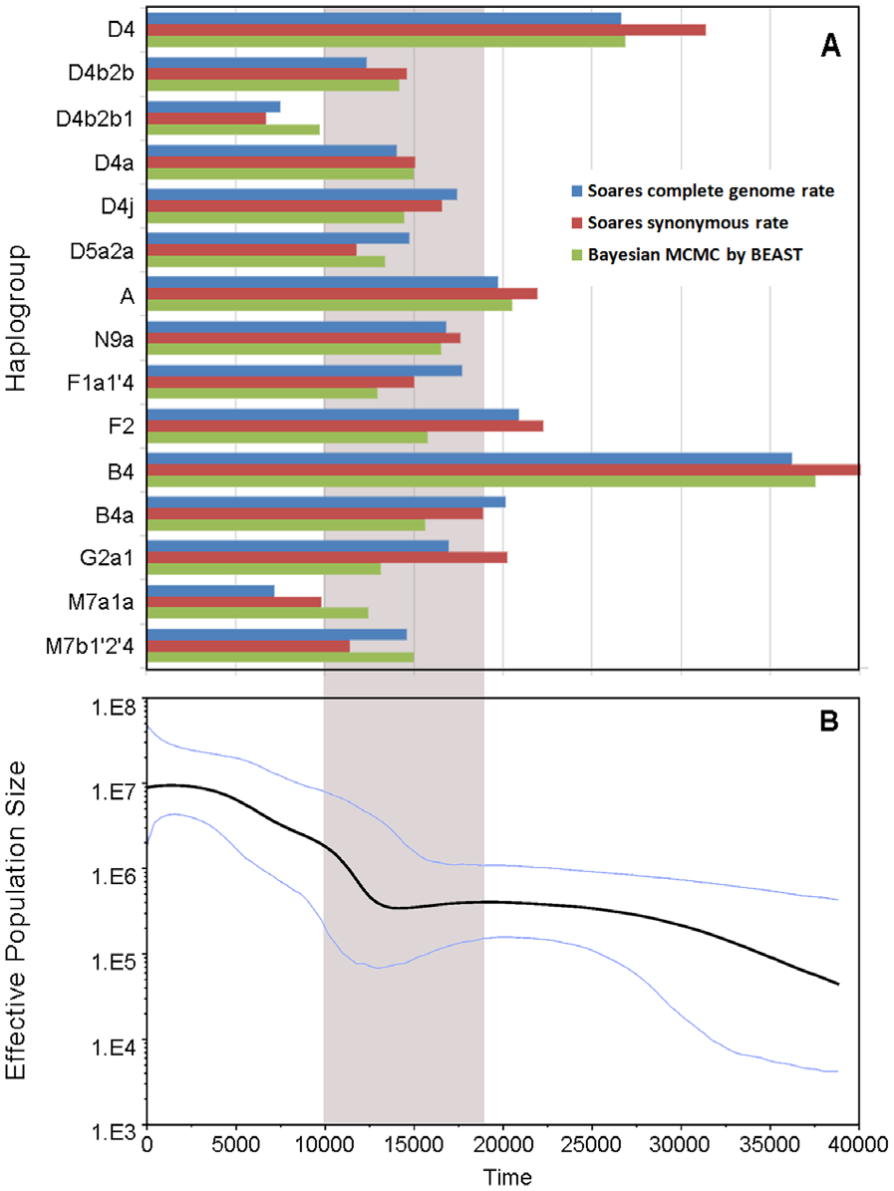
Time estimation of each observed expansion in 4 East Eurasian populations and Bayesian skyline plot for 3 Chinese populations together. (A)Time estimation of each observed expansion in 4 East Eurasian populations: ρ statistic-based method with Soares complete genome rate and Soares synonymous rate, and Bayesian MCMC method by BEAST. (B) mtDNA Bayesian skyline plot showing size trend of the 3 Chinese populations together, detailed settings refers to Methods. The grey area shows the time after LGM and before Neolithic Time.

Given the knowledge of the time of lineage expansions as shown above, we further explored the population expansions of East Asians using Bayesian skyline plot (BSP) for each of the four populations individually (Figure 2). Again, three Chinese populations showed a pre-Neolithic expansion before ∼10 kya, but JPT expanded later at ∼7kya, unequivocally during the Jōmon Period (14–2.3 kya), specifically, in the incipient Jōmon Period (8–5 kya) [10], when plant husbandry did not contribute a significant part of food source, showing a similar pattern in the mainland [27], [28]. When three Chinese populations were combined in the analysis, the BSP chart revealed a continuous expansion from ∼13 to ∼4 kya, during which the population grew by approximately 30 folds (Figure 3B). To conclude, both the estimated time of population expansion and that of lineage expansion support the occurrence of pre-Neolithic expansion in East Asia (Figure 3).

## Discussion

The three populations (CHB, CHD, and CHS) in this study, although they were collected in contemporary China, constitute an effective representation of East Asians. Based on a genome-wide analysis of SNP data, Xu et al. showed that CHB carries an assortment of genetic constituents of north (54.8%), central (13.1%), and south (32.1%) of East Asian [29] while the composition of CHD is 7.4%, 24.7%, and 67.9% [29]. The well-balanced sampling in this study should address the concern on genetic differentiation between the northern and southern populations in East Asia [30]. Furthermore, we did not observe significant difference in mtDNA haplotype frequencies between the samples of this study and the other Chinese samples in the literature [24], [25] (data not shown).

The accuracy of time estimation of lineage expansion may affect the conclusion of this study. Considering the fact that different approaches could lead to varied results [31], we employed two different methods of time estimation, i.e. the method based on ρ statistics, and the Bayesian MCMC method. The ρ-based method, is first used by Foster et al. [32], in which the accuracy of substitution rate dictates the accuracy of the result. In this analysis, two rates (complete genome rate and synonymous rate) corrected for purifying selection were used [33]. For comparison, Bayesian MCMC method, which was implemented in the program BEAST, calculates coalescence time by sampling different trees [34]. In this study, the coalescence time of each lineage estimated by the two aforementioned methods indeed showed some but not substantial discrepancies. In particular, BEAST yielded lower estimations in D4j, G2a1, F2, F1a1'4 and B4a lineages and a higher estimation in M7a1a than the ρ-based method. However, the estimations based on both methods support the conclusion that major lineage expansion in East Eurasian occurred before 10 kya. Taken together, we hypothesized that the expansions of lineages were primarily due to the expansion of populations, since the observed concomitant expansions of multiple lineages does not support the possible acts of positive selection or drift.

The onset of the Neolithic transition, i.e., the beginning of agriculture in China and Japan remains controversial among the archaeologists. As observed from Dadiwan site (7.9 kya) of Laoguantai Culture, cereal agriculture seems to be imported by the people with northern Microlithic traditions [3], indicating an earlier beginning of plant cultivation. Some recent studies traced the origin of agriculture in northern China to the Pleistocene-Holocene boundary at 10.4 kya [35], [36]. In Japan, despite of evidence for habitat management and plant domestication [9], [37], it is commonly believed that Jōmon people were foragers and gatherers. However, despite the discovery of early emergence of food production, our results clearly showed that in both China and Japan, population expansion occurred thousands of years before intensive agriculture. Therefore, agriculture should not be regarded as the prerequisite for population expansion. It is likely the other way round: the growth of population and depletion of food resources increased the cost for hunters and gatherers, and eventually triggered the intensive agriculture at late Banpo [3].

The pattern of lineage expansion also supplied another line of evidence supporting that there has been no severe and prevalent population bottleneck in East Asian since 15 kya, and even not for D4 and B4 clades before the LGM, as shown in Table 2, since they expanded in Eastern Eurasia before 20 kya. Furthermore, given the fact that D4 clades residing more likely in the northern of East Asia, we hypothesize that D4 survived the LGM, in accordance with numerous archaeological sites in North China.

Global population expansions based on mtDNA have already been investigated by several studies. Atkinson et al. [38] conducted a global Bayesian analysis on mtDNA and also did not find any major expansion after 10 kya, although they lacked mtDNA data in East Asia. Gignoux et al. [39] reported global Neolithic expansion revealed by mtDNA, by analysis on mitochondrial lineages associated with or without agriculture, also lacking data from East Asia. Gignoux et al. were not able to rule out the possibility that expansions commenced before Neolithic Time although some lineages might be associated with agriculture. Furthermore, both analyses were not based on a random sample, instead selecting mtDNA published previously and their sample sizes were relatively smaller. In our BSP results, the boundary between Neolithic and Upper Paleolithic expansions was not distinct and all the star-like clusters shared by 4 populations coalesced before 10 kya. Thus, the Neolithic expansion is likely an extension from the expansion that started at Upper Paleolithic Time.

### Conclusion

This study showed that East Asia witnessed a major population expansion that lasted for very long time (13–4 kya), based on the BSP analysis with different model settings. This expansion began at post-LGM as the temperature started to rise, i.e. before Neolithic time and the advent of agriculture. We therefore propose that the post-LGM population growth constituted a need for the introduction of agriculture, and the continuous growth of population size was likely one of the driving forces that led to the further development of agriculture and turned agriculture from a supplementary food source to a major one [40]. When the East Asian entered the Neolithic Time about 8 kya, agriculture offered the possibility of further population growth. Another possible interpretation of our results is that agriculture appeared before 8 kya, which is contradictory to the current archaeological knowledge.

## Materials and Methods

### Populations and samples

Four East Asian populations sequenced by 1000 Genome Project were included in this analysis. Southern Han Chinese (CHS) were collected from Hunan and Fujian Provinces; Beijing Han Chinese (CHB) from Beijing Normal University; Tokyo Japanese (JPT) from Tokyo metropolitan area; Denver Han Chinese (CHD) from Denver, Colorado metropolitan area. More detailed population information is listed at http://www.1000genomes.org [19]. All samples in this analysis are maternally unrelated.

### Complete mtDNA sequences assembly

The binary sequence alignment/map (BAM) files of mtDNA genomes in this study were obtained from NCBI ftp site (ftp://ftp.ncbi.nlm.nih.gov/1000genomes/). The duplicate reads were removed by MarkDuplicates, implemented in Picard v1.36 (http://picard.sourceforge.net) and the mtDNA sequences were locally realigned by GATK v1.0.4862 [41]. Pileup files were generated by SAMtools v1.0.8 [42]. Consensus sequences were then obtained based on the pileup files and indels were checked afterwards. Variations for haploid were called under the following criteria: for any single sample, the position where the mutated allele (compared with rCRS sequence) must be at least 2× coverage, and this coverage must be at least 3/4 of the total coverage on this position. If the coverage of a site is less than 2×it would be considered as a missing site, while either a mutant or its reference allele does not achieve 3/4 of the total coverage, it would be considered as a heterozygous site. Both ambiguous sites were marked with N. Finally, we obtained sequences of 367 samples (121 CHB, 73 CHD, 55 CHS, and 118 JPT) with average 1.6 ambiguous sites, and the average coverage of these 367 bams was 813× and the minimum was 8×. All the variations to rCRS were attached as supporting materials (Table S1).

### Haplogroup assignment

Complete sequences were aligned to rCRS by MUSCLE v3.8.31 [43] and manually checked, then assigned to the haplogroups according to Phylotree.org Build 10 [44], showing concordance with results from MitoTool [45]. As in Phylotree, positions 309.1C(C), 16182C, 16183C, 16193.1C(C), and 16519 were not used for haplogroup assignment since these are subject to highly recurrent mutations.

### Data analysis

The complete mtDNA median-joining network was constructed by Network v4.6 [23] using the coding region (577-16023). Each star cluster with 5 or more branches splitting out from one internal node was considered as a distinct expansion. Then, to test the assumption of a molecular clock, a maximum likelihood phylogenetic tree was also reconstructed for the coding region using PhyML v3.0 [46] under the HKY+G mutation model with an α parameter of 0.12 [47]. The null hypothesis of a molecular clock cannot be rejected (P = 1.00) using PAML package v4.4 [48]. The coalescence time of each distinct expansion was estimated using ρ statistic-based method and Bayesian MCMC method. For ρ statistic-based method, standard deviation was calculated following Saillard et al. [49]. Then the time to TMRCA of each expansion was estimated using Soares rate for synonymous mutations and for complete mitochondrial genomes (all the substitutions excluding the 16519mutation and the 16182C, 16183C, and 16194C) respectively [33]. For Bayesian MCMC analysis, the time of each distinct expansion was estimated using BEAST v1.6.1 [34]. Each MCMC sample of each cluster with distinct expansion was based on a run of 40 million generations sampled every 4,000 steps with the first 4 million generations regarded as burn-in and we combined 2 independent runs together for adequate effective sample size (>200). We used the HKY+G model of nucleotide substitution without partitioning the coding region. A strict clock was used and prior substitution rate was assumed to be normally distributed, with a mean of 2.038×10^−8^ subs/site/year and an SD of 2.064×10^−9^ subs/site/year [31]. Each run was subsequently analyzed using Tracer v1.5.1. Bayesian skyline plots for each population and 3 Chinese populations together were also generated by BEAST v1.6.1 and Tracer v1.5.1, using the similar settings as above and allowing 10 discrete changes (for each individual population) and 30 discrete changes (for 3 Chinese populations together) in the population history regarding that population size grows or declines linearly between changing points. Furthermore, for the BSP of 3 Chinese populations together, several BSP model parameters were modified to investigate the robustness of the estimation, such as molecular clock settings (relaxed or strict), discrete changes in population history (10 or 30), and population size variation between changing points (remaining constant or changing linearly). All BSP results above were similar (data not shown).

## Supporting Information

Table S1 **367 East Asian mtDNA haplotypes compared to rCRS.** Note: Sites are according to rCRS. del means deletion and ins means insertion. Others represent substitutions.
(DOC)
