# Supplementary material for: Major Population Expansion of East Asians Began before Neolithic Time: Evidence of mtDNA Genomes
Source: PLoS One. 2011 Oct 6;6(10):e25835. doi: 10.1371/journal.pone.0025835 (PMC3188578; doi:10.1371/journal.pone.0025835)
Supplement: Table S1 — 367 East Asian mtDNA haplotypes compared to rCRS. Note: Sites are according to rCRS. del means deletion and ins means insertion. Others represent substitutions. (DOC) [file pone.0025835.s001.doc]

Table S1 367 East Asian mtDNA haplotypes compared to rCRS

| HG00403 | A73G C150T T199C A263G 310insC T489C A750G A1438G A2706G G4048A C4071T A4164G A4769G A5186G A5351G G5460A A6125G C6455T T6680C C7028T T7572C T7684C G7853A C8406T A8701G A8860G T9540C T9824C A10398G C10400T T10873C G11719A C12405T C12705T T12811C C14766T T14783C G15043A G15301A A15326G G16129A C16223T T16297C |
| --- | --- |
| HG00404 | A73G G103A A263G C481T G709A A750G A1438G G1598A G2361A A2706G C4161T A4769G A4895G C7028T 8281delC 8282delC 8283delC 8284delC 8285delC 8286delT 8287delC 8288delT 8289delA G8584A C8829T A8860G G9438A T9950C T10020C A10398G G10427A A11101G G11719A A12361G A12451G C14766T T15019C C15223T A15326G C15508T A15662G T15850C A15851G G15927A A16051G C16111T G16129A T16140C G16145A A16183C T16189C C16234T T16243C A16463G T16519C |
| HG00406 | 42insG A73G C150T A263G A750G A1438G A2706G A4769N G5231A G5417A G6899A C7028T A8860G G11719A A12358G G12372A C12705T G12771A C14766T G15077A A15326G T15541N C16223T C16257A C16261T T16311C |
| HG00407 | A73G C151T A263G A479G G709A A750G A1438G A2706G C5060T C7028T T7684C T7861C A8860G A11002G G11719A G12618A G13928C C14766T A15326G T15479C T16157C T16304C |
| HG00418 | C64T A73G A210G A263G 310insC 514delC 515delA T593C G709A A750G A1438G A2706G A3537G A4769G G5237A C6960T C7028T 8281delC 8282delC 8283delC 8284delC 8285delC 8286delT 8287delC 8288delT 8289delA G8584A A8860G C9371T T9950C G10325A A10398G A10523G G11719A A11908G C14766T T14894C A15235G A15326G T16140C A16183C T16189C C16262T C16266A T16519C |
| HG00419 | C61T G62A A73G A263G C309N T310C G329A 514delC 515delA A750G A1438G G1462A C2378T A2706G A4769G C6482T C7028T 8281delC 8282delC 8283delC 8284delC 8285delC 8286delT 8287delC 8288delT 8289delA A8860G C9968T G11719A C14766T A15326G A15951G G16129A 16181delA 16182delA 16183delA T16189C G16213A T16217C C16261T C16292T C16301T T16519C |
| HG00421 | A73G T152C A263G T489C T507C A750G A1438G A2706G G3010A C3206T A4769G C4883T C5178A T5673C C7028T C8410T C8414T T8473C A8701G A8860G T9540C T9845C A10398G C10400T T10873C G11719A C12705T C13650T A13966G C14668T C14766T T14783C T14979C G15043A G15301A A15326G G15883A T15889C G16129A C16223T T16263C T16362C T16519C |
| HG00422 | A73G C150T A263G 310insC A750G A1438G A2706G A4769G G5231A G5417A C7028T A8860G T11368C G11719A A12358G G12372A C12705T C14766T T15090C G15323A A15326G T16189C C16223T C16257A C16261T A16309G |
| HG00427 | C64T A73G A210G A263G 514delC 515delA T593C G709A A750G A1438G A2706G A3537G A4769G G5237A C6960T C7028T 8281delC 8282delC 8283delC 8284delC 8285delC 8286delT 8287delC 8288delT 8289delA G8584A A8860G C9371T T9950C G10325A A10398G A10523G G11719A A11908G C14766T T14894C A15235G A15326G T16140C A16183C T16189C C16262T C16266A T16519C |
| HG00428 | A73G T195C 248delA A263G T310N T489C A750G A1438G C1715T 2226insA A2706G T3552A T4248C A4715G A4769G G6026A C7028T C7196A T7302C T7999C G8584A A8701G A8860G T9540C A9545G A10398G C10400T G10685A T10873C A10891G G11719A G11914A G11969A A12672G C12705T A13263G T14318C C14766T T14783C G15043A T15204C G15301A A15326G A15487T T15511C T15968C T16093C G16129A C16223T T16298C C16327T T16519C |
| HG00436 | A73G T152C 248delA A263G T489C A750G A1438G A2706G A4715G A4769G C5288T T5492C A5894G A6752G C7028T C7196A G8584A A8701G A8860G T9090C T9540C A10398G C10400T T10873C T10915C G11719A C12705T T14020C C14766T T14783C G15043A G15301A A15326G A15475G A15487T T15784C 15940delT C16185T T16189C C16223T C16260T T16298C A16302G |
| HG00437 | A73G 248delA A263G T310N 514delC 515delA A750G A1438G A2706G T3777C C3970T A4769G G5585A T5587C T6392C G6962A C7028T T7357C A7765G A8860G C10223T G10310A T10609C A10963G G11719A G12406A G12618A C12882T G13928C C14766T G15024A A15326G A16183C T16189C C16291T T16519C |
| HG00442 | A73G T146C C150T T195C 248delA A263G T310N T489C 514delC 515delA A750G A1438G A2706G C3970T A4769G T5587C T6392C G6962A C7028T A8860G G10310A T10609C G11719A G12406A C12882T G13928C C14766T G15024A A15326G A16183C T16189C A16300G T16304C A16305G T16311C T16519C |
| HG00443 | A73G A263G T489C A750G A1438G A2706G C2835T A3714G A4715G A4769G G6179A T6216C C7028T C7196A G8584A C8684T A8701G A8860G T9540C A10398G C10400T T10873C G11176A C11632T G11719A C12705T T14470C C14766T T14783C G15043A G15301A A15326G A15487T T15511C C16184T T16189C C16223T T16298C T16311C G16319A G16390A T16468C G16470A G16471A G16473A |
| HG00445 | A73G A263G A479G A750G C1048T A1438G A1892G A2706G C3970N C4102A C7028T T7684C T7861C A8860G A11002G G11719A G12618A G13928C C14766T A15326G T15479C T16157C T16243C T16304C |
| HG00446 | A73G T146C C150N T152N A263G A357G T471C G499A A750G A827G A1438G T1819C A2706G G4820N C7028T 8281delC 8282delC 8283delC 8284delC 8285delC 8286delT 8287delC 8288delT 8289delA A8860G G9947A G11719A G13590A C14766T A15326G C15535T T16136C T16189N T16217C T16298C T16519C |
| HG00448 | A73G C150T T199C A263G T310N T489C A750G A1438G A2706G G4048A C4071T A4164G T4598A A4769G A5351G G5460A C6228T C6455T T6680C C7028T T7684C G7853A A8701G A8860G T9540C T9824C A10398G C10400T T10873C C11407T G11719A C12405T C12705T T12811C C14766T T14783C G15043A G15301A A15326G G16129A C16223T T16297C C16527T |
| HG00449 | A73G G185A A189G T195C A263G 310insC G709A A750G A1438G A2706G A4769G T5105C C7028T T8277C 8278insN T8279C A8860G T10031C A10398G C11061T G11719A G12501A A12950G A13681G C14766T A15326G 16182delA A16183C T16189C C16270T C16278T A16299G T16311C T16362C T16519C |
| HG00452 | A73G A263G 514delC 515delA A750G A1438G A2706G A3221G G3483A C7028T G7789A 8281delC 8282delC 8283delC 8284delC 8285delC 8286delT 8287delC 8288delT 8289delA A8860G C9968T T11353C G11719A C13547T C14766T A15326G G16153A 16183delA C16184A T16189C G16213A T16217C C16261T C16292T T16362C T16519C |
| HG00457 | A73G A263G T489C A750G A1438G G2361A A2706G G3316A A4079G A5894G C7028T T8227C A8701G T8848N A8860G T9540C A10398G C10400T T10873N G11719A C12705T G14016A C14766T T14783C G15043A G15301A A15326G T15970C C16111T C16223T T16362C T16519C |
| HG00472 | A73G T152C 248delA A263G T310N 514delC 515delA G709A A750G A1438G A2706G C3970T A4769G T6392C A6599G G6962A C7028T G7805A T8772C A8860G G9053A G10310A T10454C T10609C G11719A C12360T G12406A C12882T G13759A G13928C C14766T A15326G C16111T G16129A C16266T T16304C T16519C |
| HG00473 | A73G A263G T310N T489C G709A A750G A1438G G1719A A2706G A3714G G4048A A4769G C4883T C5178A C5755N A6494G A6701G C7028T A7424G A7879G G8020A A8701G A8860G T9540C A10398G C10400T T10873C G11719A A12654G C12705T G13194A C14766T T14783C G15043A G15301A A15326G T16189C C16223T G16274A T16362C |
| HG00475 | A73G C150T A263G T489C 514delC 515delA A750G C752T T1107C A2706G C3528T A4769G C4883T C5178A A5301G C7028T A8701G A8860G A9180G T9540C A10397G A10398G C10400T T10873C G11719A T11944C A12026G C12705T C14766T T14783C G15043A G15301A A15326G T16086N T16092C A16164G T16172C 16182delA A16183C C16223T C16260T C16266T T16362C |
| HG00476 | A73G C151T T152C 248delA A263G T489C A750G A1438G A1804G A2706G C2891T A3511G A4715G A4769G A6752G C7028T T7142C C7196A G8584A A8701G A8860G T9090C T9540C A10398G C10400T T10873C G11719A C12705T C14766T T14783C G15043A G15301A A15326G A15487T T15784C 15940delT C16185T C16223T C16260T T16298C A16302G T16352C |
| HG00478 | A73G C150T T195C A263G T310N T489C 514delC 515delA A750G A1382C A1438G A2706G G3010A A4769G A4824G C4883T C5178A C7028T G8020A C8414T A8701G A8860G C8964T C9296T T9540C T9824A A10398G C10400T T10640C T10873C G11719A C12705T C14335T C14668T C14766T T14783C T15001C G15043A G15301A A15326G T16189C C16223T T16362C T16519C |
| HG00479 | A73G C150T A263G G513A G709A A750G A1438G A2706G T4386C A4769G G5231A G5417A C7028T A8860G G11719A G12007A A12358G G12372A C12705T C13578T C14766T A15326G T16093C C16111T G16129A C16223T C16257A C16261T |
| HG00512 | A73G C150T T199C T204C G207A A263G C456T T489C A750G A1438G A2706G G4048A C4071T A4164G A4769G A5351G G5460A C6455T T6680C C7028T T7684C G7853A A8701G A8860G T9540C T9824C A10398G C10400T T10873C G11719A C12405T C12705T T12811C C14766T T14783C A14978G G15043A G15301A A15326G A15515G G16129A T16189C C16223T G16274A T16297C |
| HG00513 | A73G G185A A189G A263G G709A A750G A1438G A2706G A4769G C7028T T8277N T8279N A8280N A8860G T10031C A10398G C11061T G11719A A12950G T13215C A13269G A13681G C14766T A15326G T16092C G16145A A16182C A16183C T16189C T16311C G16390A T16519C |
| HG00524 | A73G C150T T199C T204C G207A A263G C456T T489C A750G A1438G A2706G G4048A C4071T A4164G A4769G A5351G G5460A C6455T T6680C C7028T T7684C G7853A A8701G A8860G T9540C T9824C A10398G C10400T T10873C G11719A C12405T C12705T T12811C C14766T T14783C A14978G G15043A G15301A A15326G A15515G G16129A T16189C C16223T G16274A T16297C |
| HG00525 | A73G C150T T199C A263G T310N T489C A750G A1438G A2706G G4048A C4071T A4164G G4580A A4715G A4769G G5262A A5351G G5460A A6266G C6455T T6680C C7028T T7684C G7853A A8701G A8860G T9540C T9824C A10398G C10400T T10873C G11719A C12405T C12705T T12811C C14187T C14766T T14783C G15043A G15110A G15301A A15326G G16129A C16192T C16223T T16297C |
| HG00530 | A73G T146C 248delA A263G 514delC 515delA G709A A750G A1438G C1734T A2706G C3970T A4722G A4769G T5628C T6392C G6962A C7028T T7738C T8705C A8860G G10310A T10609C G11719A T11935C G12406A C12858T C12882T G13928C C14766T A15326G C15402T T16189C T16304C T16519C |
| HG00531 | A73G C150T T152C A263G T310N A750G A1438G G1664A A2706G A4769G G5231A G5417A C7028T A8860G A9156G G11719A A12358G G12372A C12705T C14766T A15326G T16092C G16145A T16172C C16223T C16245T C16257A C16261T T16519C |
| HG00536 | A73G G185A A189G A234G A263G A470G G709A A750G A1438G A2706G A4769G A4775G C7028T T8277C 8278insN T8279C A8860G T10031C A10398G C11061T G11719A A12950G A13269G A13681G C14766T A15326G A16182C A16183C T16189C T16311C G16390A T16519C |
| HG00537 | A73G T152C A193G A263G T310C 514delC 515delA G709A A750G A1438G A2706G A4769G T5201C T5465C C7028T 8281delC 8282delC 8283delC 8284delC 8285delC 8286delT 8287delC 8288delT 8289delA A8860G G9123A G11719A C14751T C14766T A15326G A16182C A16183C T16189C T16217C C16223T A16299G T16519C |
| HG00542 | A73G A210G A263G 514delC 515delA T593C G709A A750G A1438G A2706G A3537G A4769G G5237A C6960T C7028T 8281delC 8282delC 8283delC 8284delC 8285delC 8286delT 8287delC 8288delT 8289delA G8584A A8860G T9950C G10325A A10398G A10523G G11719A C14766T A15235G A15326G T16140C A16182C A16183C T16189C C16266A T16519C |
| HG00543 | A73G T146C A200G A210G C262T A263G 514delC 515delA G709A A750G A1438G A2706G A3537G A4769G C7028T 8281delC 8282delC 8283delC 8284delC 8285delC 8286delT 8287delC 8288delT 8289delA G8584A A8860G T9950C G9962A A10388G A10398G C11151T G11719A C14766T A15235G A15326G A15671G C16188T T16189C C16266G C16278T T16519C |
| HG00556 | A73G C150T T195C A263G T453A A750G 955insN T961C A1438G A2706G A4769G G5231A G5417A C5755N T6092C C7028T G7598A A8860G A9180G G10801A G11719A A12358G G12372A C12705T C13934T T14581C C14766T A15326G T16172C C16223T C16257A C16261T T16311C T16519C |
| HG00557 | A73G A235G A263G T310C 514delC 515delA A663G A750G T961C A1438G G1709A A2706G T4248C A4769G A4824G G6366A C7028T G8251A A8563G C8794T A8860G C11536T G11719A C12705T C14766T A15326G G15355A T16126C C16223T C16234T A16235G C16290T G16319A T16519C |
| HG00559 | A73G A263G 310insC T489C 567insC 567insC 567insC G709A A750G A1438G A2706G 3167insC T4023C C4140T A4769G C7028T A7250G T7660C A8701G T8793C A8820G G8856A A8860G T9540C A9964N A10398G C10400T G10646A T10873C G11719A C12549T C12705T A13152G A14128G T14502C C14766T T14783C C15040T G15043A T15071C A15218G G15301A A15326G A15769G A16066G T16092C C16223T T16311C |
| HG00560 | A73G 248delA G251A A263G 514delC 515delA A750G A1438G A2706G C3970T C4086T A4769G T4967C T6392C G6962A C7028T A8860G G9053A G9548A G10310A T10463C T10609C G11719A G12406A G12630A C12882T G13759A G13928C C14766T A15326G G16129A A16162G T16172C T16304C A16335G T16519C |
| HG00565 | A73G T152C A235G A263G T310N 514delC 515delA A663G A750G A1438G A1736G A2706G T4248C T4314C A4769G A4824G C7028T A7298G A8531G C8794T A8860G G11719A C12705T C14766T A15326G T16093C C16179A C16223T C16290T G16319A |
| HG00566 | A73G T152C A235G A263G 514delC 515delA A663G A750G A1438G A1736G A2706G T4248C A4769G A4824G A5512G C7028T C8794T A8860G G10646A G11719A C12705T G13590A C14766T A15326G C16223T C16290T G16319A |
| HG00577 | A73G C150T A263G T310N A750G A1438G A2706G A4129G G4512A A4769G G5231A G5417A C7028T A8860G G11719A T12354C A12358G G12372A A12612G C12705T C13890T C14766T G15106A A15326G G16129A C16223T C16257A C16261T |
| HG00578 | A73G T146C A263G C371T T489C A750G A1438G A2706G G3010A C3746T A4769G C4883T C5178A T6176C C7028T C8414T A8701G A8860G T9540C A10398G C10400T T10873C A11239G G11696A G11719A T11878C C12705T T13020C G13477A C14668T C14766T T14783C G15043A G15301A A15326G C15529A C16188T T16209C C16214T C16223T T16362C |
| HG00592 | A73G C150T A263G T310N A750G A1438G A2706G T4386C A4769G G5231A G5417A C7028T A8860G G11719A G12007A A12358G G12372A C12705T C14766T A15326G C16111T G16129A C16223T C16257A C16261T |
| HG00593 | A73G C150T T199C A263G T489C A750G T980C A1438G G1503A A2706G G4048A C4071T A4164G A4769G A5351G G5460A A6125G C6455T T6680C C7028T T7684C G7853A A8701G A8860G T9540C T9824C A10398G C10400T T10873C G11719A C12405T C12705T T12811C C14766T T14783C G15043A G15301A A15326G G16129A C16192T C16223T T16297C T16519C |
| HG00596 | A73G A263G T310C G709A A750G T1119C A1438G A2706G C3497T A4769G C7028T 8281delC 8282delC 8283delC 8284delC 8285delC 8286delT 8287delC 8288delT 8289delA A8860G G10310A G11719A A12366G A14133G C14766T A15326G G15346A A16183C T16189C T16217C T16311C T16519C |
| HG00610 | A73G G103A A263G T310N 514delC 515delA G709A A750G 955insC A1438G G1598A A2706G T3898N A4769G C7028T A7822G 8281delC 8282delC 8283delC 8284delC 8285delC 8286delT 8287delC 8288delT 8289delA G8584A A8784G C8829T A8860G G9053A T9950C A10398G G11719A A11928G A12361G C14632T C14766T C15223T A15326G C15508T A15662G A15851G G15927A T16140C A16183C T16189C T16243C T16519C |
| HG00611 | A73G T146C T199C A263G T310N T489C 514delC 515delA A750G A1438G A2706G G3882A C4071T A4769G C4850T T5442C C6455T C7028T A8701G A8860G T9540C T9824C T9833C A10398G C10400T T10873C C11665T G11719A T12091C G12372A C12705T A12810G G13759A T14308C C14766T T14783C G15043A G15301A A15326G C16223T C16278T C16295T T16519C |
| HG00625 | A73G C194T A263G T489C 514delC 515delA A750G A1382C A1438G A2706G G3010A A4769G C4883T C5178A C7028T G8020A C8414T A8701G A8860G C8964T C9296T T9540C T9824A A10398G C10400T T10873C G11719A C12705T T13500C G13590A C14668T C14766T T14783C G15043A G15301A A15326G A15896G T16209C C16223T C16266T T16362C T16519C |
| HG00626 | A73G T125C T127C C128T A263G T310N T318C T489C G513A 514delC 515delA A750G A1438G A2706G C4170T A4769G T5580C C7028T G7269A T8654C A8701G A8860G C9490T T9540C A10398G C10400T T10873C G11719A A12030G A12358G G12372A C12705T G14569A T14727C C14766T T14783C A15010G G15043A G15301A A15326G A15463G C15651T C16223T C16234T C16287T C16290T T16362C |
| HG00628 | A73G C150T A263G T310C T489C 514delC 515delA 567insC A750G C752T T1107C A2706G A4769G C4883T C5178A A5301G A6779G C7028T A8701G A8860G A9033G A9180G T9540C G9554A A10397G A10398G C10400T T10873C G11719A T11944C A12026G C12705T C14766T T14783C G15043A G15301A A15326G T16092C A16164G 16182delA 16183delA T16189C C16223T C16266T T16362C |
| HG00629 | A73G C298T T489C A750G A1438G A2706G G3010A C4394T A4769G C4883T C5178A G5231A T5655C C7028T C8414T A8701G A8860G A9007G T9540C A10398G C10400T T10873C C11059T G11719A G12372A C12705T A13104G C14668T C14766T T14783C G15043A A15085G G15301A A15326G C16169T C16223T G16274A T16311C T16362C |
| HG00634 | A73G T152C A235G A263G 514delC 515delA A663G A750G A1438G A1736G A2706G A3891G T4248C A4769G A4824G C7028T A8440G C8794T A8860G T8939C T11465C G11719A C12705T A12975T C14766T A15326G C16223T C16290T G16319A T16362C |
| HG00635 | A73G T146C A263G T310N C371T T489C A750G A1438G A2706G G3010A C3746T A4769G C4883T C5178A T6176C C7028T C8414T A8701G A8860G T9540C A10398G C10400T T10873C A11239G G11696A G11719A T11878C C12705T T13020C G13477A C14668T C14766T T14783C G15043A G15301A A15326G C15529A C16188T T16209C C16214T C16223T T16362C |
| NA17962 | A73G C150T A263G T489C A750G C752T T1107C A1438G A2706G C3212T A4769G T4823C C4883T C5178A A5301G C7028T A8701G A8860G A9180G T9540C A10397G A10398G C10400T T10873N C11257T G11719A T11944C A12026G C12705T G13759A C14766T T14783C G15043A G15301A A15326G A16182N A16183C T16189C C16223T C16360T T16362C |
| NA17963 | A73G 248delA A263G 514delC 515delA A750G A1438G A2706G C3970T C4086T T4695C A4769G T6392C G6962A C7028T T7372C A8860G G9053A G10310A T10604C T10609C G11719A G12406A C12882T G13759A G13928C C14766T A15326G G16129A A16141G T16172C T16304C T16519C |
| NA17965 | A73G A153G A263G T489C A750G A1041G A1438G A2706G T3394C T4216C G4491A A4769G C7028T T7142C T7861N A8701G A8860G A9242G T9540C A10398G C10400T T10873N G11719A C12705T T14308C A14417G C14766T T14783C G15043A G15301A A15326G T16092C C16174N C16223T C16234T C16291T A16316G T16362C |
| NA17967 | A73G T152C A235G A263G 514delC 515delA A663G A750G A1438G A1736G A2706G T4248C A4769G A4824G T6378C C7028T C8393T C8794T A8860G G9123A T9128C A9150G G11719A A12200G C12705T C14766T A15326G C16223T C16290T G16319N T16362C |
| NA17969 | A73G T199C A263G T489C A750G A1438G G1664A A2706G G4048A C4071T A4164G A4769G A5351G G5460A T6351C C6455T T6680C C7028T T7684C G7853A A8701G A8860G A9468G T9540C T9824C A10398G C10400T C10497T T10873C G11719A T12121C C12405T C12705T C14115T C14766T T14783C G15043A G15301A A15326G G15812A T16086C G16129A T16297C T16519C |
| NA17971 | A73G C150T T199C T204C A263G A374G T489C C509T A750G A1438G A2706G G4048A C4071T A4164G A4769G A5351G G5460A C6455T T6680C C7028T T7684C G7853A A8701G A8860G T9540C T9824C A10398G C10400T T10873C G11719A C12405T C12705T T12811C T13401C T14256C C14766T T14783C G15043A G15301A A15326G G16129A C16192T C16223T C16291T T16297C T16519C |
| NA17976 | A73G A263G T489C G709A A750G A1438G A2706G A4769G A4833G T5108C G5460A T6896C A8701G A8860G T9540C A10398G C10400T T10873N G11719A C12705T G13477A G14569A A14605G C14766T T14783C G15043A G15301A A15326G G15927A T16093C T16209C C16223T G16274A A16309G T16362C |
| NA17977 | G53A G54C 66delG A73G 248delA A263G T318C 514delC 515delA A750G A1438G A2706G C3970T C4086T A4769G T6392C G6962A C7028T A8718G A8860G G9053A G10310A T10609C G11719A G12406A C12882T C13044T G13759A G13928C C14766T A15326G T15565C G16129A T16172C T16189C T16304C |
| NA17979 | A73G T146C C150T A263G G709A A750G T1119C A1438G A2706G C3497T C3571N A4769G C7028T 8281delC 8282delC 8283delC 8284delC 8285delC 8286delT 8287delC 8288delT 8289delA T8772C A8860G A9389G A11293G G11719A A13105G C14766T G15301A A15326G G15346A T16140C A16182C A16183C T16189C T16217C G16274A A16335G T16519C |
| NA17980 | A73G C151T T152C 248delA A263G T489C A750G A1438G A2706G A4715G A4769G A6752G C7028T C7196A G8584A A8701G A8860G T9090C T9540C A10398G C10400T T10873N G11719A C12705T C14766T T14783C G15043A G15301A A15326G A15475G A15487T T15784C 15940delT C16185T C16223T C16260T T16298C A16302G |
| NA17981 | A73G A263G T489C A750G A1438G A2706G G3010A G3316A A4385G A4769G C4883T C5178A C7028T C8414T A8701G A8860G C9536T T9540C A10398G C10400T T10873C C11215T G11719A C12705T C14668T C14766T T14783C G15043A G15301A A15326G T16092C C16223T T16362C T16519C |
| NA17982 | A73G T195C 248delA A263G T489C A750G A1438G A2706G T3552A A4715G A4769G A5573G G5821A T5918C A6338G C7028T C7196A A8233N G8584A A8701G A8860G T9540C A9545G A10398G C10400T T10873C A11101G G11719A G11914A A11923G C12705T T14318C C14766T T14783C G15043A G15301A A15326G A15487T G15928A T16209C C16223T T16298C G16319A C16327T T16519C |
| NA17983 | A73G C150T A200G A215G A263G T318C A326G T471C T489C A750G T1095C A1438G A2706G A3523G T4768C A4769G C5486T G6383A C6531T C7028T G7642A A8108G A8701G A8860G T9540C T9950C A10398G C10400T T10873C A11350G G11719A G11969A C12705T C13272A C14766T T14783C T14788C G15043A G15301A A15326G T16172C C16223T C16295T T16519C |
| NA17986 | A73G A263G T489C A750G A1438G A2706G G3010A G3316A A4385G A4769G C4883T C5178A C7028T C8414T A8701G A8860G C9536T T9540C A10398G C10400T T10873C C11215T G11719A C12705T C14668T C14766T T14783C G15043A G15301A A15326G T16092C C16223T T16362C T16519C |
| NA17987 | A73G A235G A263G 514delC 515delA A663G A750G 955insN T961C A1438G G1709A A1736G A2706G T4248C A4769G A4824G C7028T T8260C A8563G C8794T A8860G C11536T G11719A C12705T C14766T A15326G T16126C C16223T A16235G C16290T G16319A T16519C |
| NA17988 | A73G A153G 248delA A263G 514delC 515delA A750G T1005C A1438G T1824C A2706G C3970T A4769G A4811G T5090C T6392C T6782C C7028T A7828G A8860G G10310A T10535C G10586A G11377N G11719A T12338C G13708A G13928C C14766T A15326G T16092A C16291T T16304C |
| NA17989 | A73G A200G 248delA A263G 514delC 515delA A750G A1438G A2706G C3970T C4086T A4769G T6392C G6962A C7028T A8860G G9053A G10310A T10609C G11719A G12406A G12501A C12882T G13759A G13928C G14259A C14766T A15326G T16172C T16304C T16519C |
| NA17990 | A73G G94A T236C A263G 514delC 515delA A750G A1438G A2706G A4769G T5465C C7028T 8281delC 8282delC 8283delC 8284delC 8285delC 8286delT 8287delC 8288delT 8289delA A8860G G9123A G11719A C12239T C14766T A15326G G16129A A16182N A16183C T16189C T16217C C16261T T16519C |
| NA18103 | A73G T152C A193G A263G G499A A750G A827G A1438G A2706G A4769G G4820A C5129T C7028T G7521A 8281delC 8282delC 8283delC 8284delC 8285delC 8286delT 8287delC 8288delT 8289delA A8860G T9101G C9727T A11239G G11719A G11914A A12242N G13590A A14587G C14766T A15326G C15535T T16136C A16183N T16189N T16217C C16218T T16519C |
| NA18105 | A73G A263G T489C A750G A1438G A2706G C2835T A4715G A4769G G6179A C7028T C7196A G8584A C8684T A8701G A8860G T9540C A10398G C10400T T10873C G11719A C12705T T14470C A14641G C14766T T14783C G15043A G15217A G15301A A15326G A15487T C16184T C16223T T16298C G16319A |
| NA18106 | A73G T146C C150T C151T T152C C182T T217C A263G T489C A750G T1107C A1438G A2706G C3546T A4200T T4216C G4659A A4769G C4883T C5178A G5261A A5301G C7028T A8701G A8860G T9540C A10397G A10398G C10400T T10873N G11719A C12705N A13105G A14696G C14766T T14783C A14927G G15043A T15128C G15301A A15326G C15529T T15622C T16189N T16362C T16519C |
| NA18107 | A73N 248delA A263G 514delC 515delA A750G A1438G A2706G C3970T C4086T A4769G T6392C G6962A C7028T A8860G G9053A G9554A T9944C G10310A T10463C T10609C G11719A G12406A C12882T A13748G G13759A G13928C G14040A C14766T A15326G T15565C G16129A T16172C T16304C T16311C T16519C |
| NA18108 | A73G A263G T489C A750G A1438G A2706G G3010A A4395G A4769G C4883T T5021C C5178A C7028T C8414T A8701G A8860G T9540C A10398G C10400T T10873N C11215T G11719A C12705T G14384A C14668T C14766T T14783C G15043A G15106A T15184C G15301A A15326G C16223T C16234T G16274A C16291T T16362C |
| NA18109 | A73G A263G T267C 514delC 515delA A750G A1438G T1658C A2706G A4769G G5703A C7028T G7789A 8281delC 8282delC 8283delC 8284delC 8285delC 8286delT 8287delC 8288delT 8289delA G8485A A8860G C9968T T11353C G11719A C13547T C14766T A15326G T16093C G16153A A16181N A16182N 16183delA T16189C G16213A T16217C C16261T C16292T T16519C |
| NA18110 | A73G C150T T152C A263G G499A A750G A827G A1438G T1819C A2706G A4769G G4820A C6482T C7028T 8281delC 8282delC 8283delC 8284delC 8285delC 8286delT 8287delC 8288delT 8289delA A8860G A9377G G11719A G13590A C14766T A15326G C15535T T16136C A16183C T16189C T16217C C16270T T16298C T16519C |
| NA18111 | A73G C150T A263G G709A A750G T1119C A1438G A2706G C3435T C3497T C3571T A4769G C7028T 8281delC 8282delC 8283delC 8284delC 8285delC 8286delT 8287delC 8288delT 8289delA A8860G G8994A T9128C G11440A G11719A C14766T A15326G G15346A T16140C A16183N T16189N T16217C G16274A A16335G T16519C |
| NA18112 | A73G 248delA A263G 514delC 515delA C548T A750G A1438G A2706G T3645C C3970T C4086T A4769G T6392C G6962A C7028T A8860G G9053A G9548A C10211N G10310A T10609C G11719A G11969A G12406A C12882T G13759A G13928C T14325C C14766T A15326G G16129A A16162G T16172C G16274A T16304C T16311C C16320T T16519C |
| NA18113 | A73G C150T C151T T152C A200G A263G 310insC T489C A750G T1107C A1438G A2706G G3316A A4200T T4216C A4769G C4883T C5178A A5301G C7028T A7129G C7669T C8580T A8701G A8860G T9540C A9545G A10397G A10398G C10400T T10873C G11719A C12705T A12810G C13984T C14766T T14783C A14927G G15043A G15301A A15326G T15622C G15737A C16079T C16223T T16311C A16316G T16362C |
| NA18115 | A73G A263G T489C A750G A1041G A1438G A2706G T3394C A3873G G4491A A4769G C7028T A8701G A8860G A9242G T9540C A10398G C10400T T10873C G11719A G11963A C12705T G12772A A13731G T14308C C14766T T14783C G15043A G15301A A15326G C16223T C16234T A16316G T16362C |
| NA18116 | A73G A263G 310insC T489C G709A A750G A1438G A2706G A4769G A4833G T5108C G5460A T6896C A8701G A8860N T9540C A10398G C10400T T10873C G11719A C12705T G13477A G14569A A14605G C14766T T14783C G15043A G15301A A15326G G15927A T16093C T16209C C16223T G16274A A16309G T16362C |
| NA18117 | A73G A263G G316N A750G A827G A1438G A2706G A4769G C7028T C7864T 8281delC 8282delC 8283delC 8284delC 8285delC 8286delT 8287delC 8288delT 8289delA A8860G G11719A G11914A T12732C A13942G C14766T A15038G A15326G C15535T G15930A C16176T A16183N T16189C T16217C T16519C |
| NA18119 | A73G T146C C150T 248delA A263G 310insC 514delC 515delA A750G A1438G A2706G C3970T C4086T A4769G T6392C G6962A C7028T A8149G A8860N G9053A G9548A G10310A T10609C G11719A G12406A C12882T G13759A G13928C C14766T A15326G C16108T A16162G T16172C T16304C T16519C |
| NA18122 | A73G T204C G207A 248delA A263G G709A A750G A1438G C1721N A2706G G2817N C2818N T2871N A3434G C3970T A4769G G5585A A5894G G5913A A5978G T6392C C7028T A8860G T9854C G10310A G10320A A10499G A11065G G11719A C12621T G13928C C14766T T14971C A15326G T15943C T16140C C16260T T16298C C16355T T16362C |
| NA18123 | A73G C150T T204C G207A A263G 310insC G499A A750G A827G A1438G G1719A A2220G A2706G A4769G G4820A 5894insC 5894insC T6216C T6413C C7028T A8860G G11719A G13590A C14766T A15326G C15535T T16136C T16189N T16217C T16519C |
| NA18124 | A73G C150T T199C A263G T489C A750G A1438G A2706G G4048A C4071T A4164G A4769G A5351G G5460A C6228T C6455T T6680C C7028T T7684C G7853A A8701G A8860G T9540C T9824C A10232G A10398G C10400T T10873C G11719A C12405T G12561A C12705T T12811C C14766T T14783C G15043A G15301A A15326G G16129A C16192T C16223T T16297N |
| NA18126 | G1N A2N T3N C4N A73G T146C T152C T199C A263G 310insC T489C 514delC 515delA A750G A1438G A2706G C4071T A4769G C4850T T5442C C6455T C7028T A7091N G7337A A8701G A8860N T9103C T9540C T9824C T9957C A10398G C10400T T10861C T10873C C11665T G11719A T12091C G12561A C12705T G13590A C14766T T14783C G15043A G15301A A15326G C16223T C16295T T16297C T16519C C16565N G16566N A16567N T16568N G16569N |
| NA18127 | A73G 248delA A263G T489C A750G A1438G 2226insA A2706G T3552A T4254C A4715N A4769G A4877N C4883N G5821A A6338G C7028T C7196A G7853A G8584A A8701G A8860G T9540C A9545G A10398G C10400T T10873C G11719A G11914A C12705T A13263G T14318C C14766T T14783C G15043A G15301A A15326G A15487T T16189C C16223T T16298C C16327T T16519C |
| NA18130 | A73G C151T T152C A263G G499A 514delC 515delA A750G T980C A1438G A1811G A2706G C3741T A4769G C5360N C7028T C8137T C8684T A8860G A9852G C10142T A11467G G11719A A12308G G12372A G12618A T13500C G14569A C14766T A15326G A16318T T16519C |
| NA18132 | A73G T152C 248delA A263G 514delC 515delA 516delC 517delA A750G A1438G A2706G C3970T C4086T A4769G G5773A A5978G G5985A T6392C G6962A C7028T A8860G G9053A G9548A G10310A T10609C G11719A C11857N G12406A C12882T G13759A G13928C C14766T A15326G T15445C G16129A T16172C T16304C T16311C C16360T T16362C T16519C |
| NA18133 | A73G G185A A189G T195C A259G A263G A508G G709A A750G A1438G A2706G A4769G C7028T T8277C 8278insC T8279C A8502N A8860G T10031C A10398G C11061T G11719A A12950G A13269G A13681G C14766T A15326G A16182N A16183N T16189C T16311C T16519C |
| NA18134 | A73G T146C A263G A750G A1438G A2706G G3834A A4769G G5417A C7028T G8392A A8860G C8996N T9078N C9278T 9532delC A10097G A10398G G11719A C12705N T13743N T14178C A14693G C14766T G15221A A15326G C15460N T16126C T16231C C16266T T16519C |
| NA18138 | A73G T146A T199C A263G T489C 514delC 515delA A750G A1438G A2706G A3606G C4071T A4769G C4850T T5442C G6249A C6455T C7028T A8701G A8860G T9540C T9824C A10398G C10400T T10873C C11665T G11719A T12091C C12705T C14766T T14783C G15043A A15236G G15301A A15326G T16362C T16519C |
| NA18140 | A73G T152C A235G A263G 513insC 513insA A663G A750G A1438G A1736G A2706G T4232C T4248C A4769G A4824G T6827C C7028T C8794T A8860G G10320A T10335C G11719A C12705T C14766T A15326G C16223T C16290T G16319A T16362C |
| NA18141 | A73G A183G A263G 514delC 515delA A750G A1438G A2706G A4769G G5231A A6485G C7028T G7789A 8281delC 8282delC 8283delC 8284delC 8285delC 8286delT 8287delC 8288delT 8289delA A8860G C9488T C9968T G11719A G11963A A13167G T13191C T13488C C14766T A15326G G15346A A16181N A16182N A16183C T16189C G16213A T16217C C16261T C16292T T16519C |
| NA18145 | A73G C194T A263G 310insC T489C 514delC 515delA A750G A1382C A1438G A1978G A2706G G3010A A3796G A4769G C4883T C5178A T5291C C7028T G8020A C8414T A8701G A8860G C8964T C9296T T9540C T9824A A10398G C10400T T10873C G11719A C12705T T13182C C14668T C14766T T14783C G15043A G15301A A15326G C15832T C16223T T16243C T16362C T16519C |
| NA18146 | A73G T195C A200G A235G A263G 514delC 515delA A663N A750G A1438G A1736G 2151delA A2706G T4248C A4769G A4824G C7028T C8794T A8860G T10084C A10750G G11447A G11719A G12236N C12705T G13590A C14766T A15326G G16129A C16223T C16290T G16319A T16362C T16519C |
| NA18149 | A73G C150T T199C A263G T489C T593C A750G A1438G T1694C A2706G G4048A C4071T C4137T A4164G A4769G A5351G G5460A C6455T T6680C C7028T T7684C G7853A A8701G A8860G T9540C T9824C T10361C A10398G C10400T T10873N C11659T G11719A C12405T C12705T T12811C C14766T T14783C G15043A G15301A A15326G G16129A T16189C C16223T T16297N T16519C |
| NA18151 | A73G T152C A263G T489C 513insC 513insA A750G A1438G T1520C A2706G G3010A C3206T A4769G C4883T C5178A A5466G C7028T G7912A C8414T T8473C A8701G A8860G T9540C A10398G C10400T T10873C G11719A C12705T T12892C A13834G C14668T C14766T T14783C T14979C G15043A G15301A A15326G C15481T G16129A C16223T T16249C C16278T T16311C T16362C |
| NA18152 | A73G T146C T199N A263G T489C 514delC 515delA A750G A1438G A2706G C4071T A4769G C4850T T5442C C6455T C7028T G7337A A8701G A8860G T9540C T9824C T9957C A10398G C10400T T10861C T10873C C11665T G11719A T12091C G12561A C12705T G13590A C14766T T14783C G15043A G15301A A15326G C16167T C16223T C16295T T16519C |
| NA18155 | A73G T146C 248delA A263G T489C A750G A1438G A2706G T3552A A4715N A4769G G5821A A6338G C7028T C7196A G7853A G8584A A8701G A8860G T9540C A9545G A10398G C10400T T10873C G11719A G11914A C12705T T12957C A13263G T14318C C14766T T14783C A14978G G15043A G15301A A15326G A15487T C16223T T16231C T16298C C16327T T16519C |
| NA18156 | A73G C150T T195C A263G G709A A750G T1119C A1438G A2706G C3497T C3571T A4769G C7028T T8200C 8281delC 8282delC 8283delC 8284delC 8285delC 8286delT 8287delC 8288delT 8289delA T8634C A8860G A10157G G11719A C14766T A14793G A15326G G15346A T16140C A16183N T16189C T16217C G16274A A16305C A16335G T16519C |
| NA18157 | A73G A263G T489C A750G A1438G A2706G C2835T A4715G A4769G G6179A C7028T C7196A G8584A C8684T A8701G A8860G T9540C A10398G C10400T T10873C G11719A C12705T T14470C C14766T T14783C G15043A G15301A A15326G A15487T C16184N C16223T T16298C G16319A |
| NA18160 | A73G 248delA A263G T489C A750G A1438G A2706G T3552A T4254C A4715G A4769G G5821A A6338G C7028T C7196A G7853A G8584A A8701G A8860G T9540C A9545G A10398G C10400T T10873C G11719A G11914A C12705T A13263G T14318C C14766T T14783C G15043A G15301A A15326G A15487T T16189N C16223T T16298C C16327T T16519C |
| NA18161 | A73G A263G T489C G709A A750G A1438G A2706G A4769G A4833N T5108C C5601T C7028T G7600A A8701G A8860G A9377G T9540C G9575A A10398G C10400T T10873N T11087C G11719A C12705T A13563G T14200C G14569A C14766T T14783C G15043A G15172A G15301A A15326G G16129A C16223T C16278T T16362C |
| NA18163 | A73G C150T A263G T489C G709A A750G A1438G A2706G A3447G G3834A A4769G A4833G T5108C T5806C T6842C C7028T A7772G C7867T T8200C A8701G A8860G T9540C A10398G C10400T T10873C G11719A C12705T G14569A C14766T T14783C G15043A G15301A G15323A A15326G T15462C G15497A A15860G T16189N C16221T C16223T T16325C T16362C T16519C |
| NA18164 | A73G T152C A235G A263G A663G A750G A1438G A1736G A2706G T4248C A4769G A4824G C7028T C8794T A8860N C8861N A8862N G11719A C12705T A14696G C14766T A15326G C16148T C16223T C16290T G16319A T16362C |
| NA18166 | A73G T152C G207A A235G 514delC 515delA A663G A750G A1438G A1736G A2706G T4248C A4769G A4824G C7028T A8459G C8794T A8860G A11084G G11719A C12705T C14067T C14766T A15326G C16223T C16290T G16319A |
| NA18525 | A73G T152C T195C 248delA A263G G275A A750G T1005C A1438G T1824C A2706G T3753C C3970T A4769G T6392C C7028T A7828G A8860G T10084C G10310A T10535C G10586A C11323T T11410C G11719A T12338C G13708A G13928C C14766T A15326G C16221T T16304C T16519C |
| NA18526 | A73G G260A A263G T489C G709A A750G A1438G A2706G A4769G T5108C C5601T A6737G C7028T G7600A A8108G A8440G A8701G C8748T A8860G C9105T A9377G T9540C G9575A T9935C A10398G C10400T T10873C G11719A T12311C C12705T A13563G T14200C G14569A C14766T T14783C G15043A G15301A A15326G C16223T C16278T T16362C |
| NA18527 | A73G A263G T489C A750G A1438G G1709A A2706G G3010A A4769N C4883T C5178A C7028T T7220C C8414T A8701G A8860G T9540C A10398G C10400T T10873C G11696A G11719A C12705T C14668T C14766T T14783C G15043A T15139N G15301A A15326G C16184T C16223T T16311C T16362C |
| NA18528 | A73G A263G 514delC 515delA A750G A1438G A2706G A4769G T5465C T6620C A6890G C7028T T7278C 8281delC 8282delC 8283delC 8284delC 8285delC 8286delT 8287delC 8288delT 8289delA A8860G G8950A G9123A C9812T G11719A C14766T A15326G A16182C A16183C T16189C T16217C C16256T C16261T A16326G C16360T T16519C |
| NA18531 | A73G C150T A263G T489C 514delC 515delA A750G C752T T1107C A2706G A4769G C4883T C5178A C5263T A5301G T6671C C7028T A8701G A8860G A9180G T9540C G10143A A10397G A10398G C10400T T10873N G11719A T11944C A12026G C12705T C14766T T14783C G15043A G15301A A15326G C16111T A16164G T16172C A16182N A16183C T16189C C16223T C16266T T16362C T16519C |
| NA18532 | A73G A263G T489C A750G A1222G A1438G G1719A A2706G G3010A A3492G A4767G A4769G C4883T C5178A C7028T C8414T A8701G T8762C A8860G T9540C A10398G C10400T T10873C G11719A G12651C C12705T T14180C C14668T C14766T T14783C G15043A G15301A A15326G G16042A C16214T C16223T T16311C T16362C |
| NA18533 | A73G C150T A263G T489C 514delC 515delA A750G C752T T1107C A2706G A4769G C4883T C5178A A5301G C7028T G8251A A8701G A8860G A9180G T9540C A10397G A10398G C10400T T10873C G11719A T11944C A12026G C12705T C14766T T14783C G15043A G15301A A15326G T16092C A16164G T16172C A16183N T16189C C16223T C16266T T16362C |
| NA18535 | A73G A263G 310insC T489C 514delC 515delA A750G C1001A A1438G A2706G G3010A A4769G C4883T C5178A A6881G C7028T G8020A C8414T A8701G A8860G G9142A T9540C C10181T A10398G C10400T T10873C G11719A C12705T C13720T C14668T C14766T T14783C C14815T A14927G G15043A G15301A A15326G T15440C A15951G C16223T T16311C G16319A T16362C |
| NA18537 | A73G A193G A263G 514delC 515delA G709A A750G A1438G A2706G A4769G T5465C A5498G C7028T 8281delC 8282delC 8283delC 8284delC 8285delC 8286delT 8287delC 8288delT 8289delA A8860G G9123A G11719A A13834G C14751T C14766T T15262C A15326G A16182N A16183C T16189C T16217C A16299G T16519C |
| NA18538 | A73G A263G T489C A750G A1438G A2706G C2835T A4715G A4769G G6179A C7028T C7196A G8584A C8684T A8701G A8860G T9540C A10398G C10400T T10873C G11719A C12705T T14470C C14766T T14783C G15043A G15301A A15326G A15487T C16184T C16223T T16298C G16319A |
| NA18539 | A73G C150T T195C A235G A263G C560T A750G A799G A1438G A1811G A2706G A3480G A4769G C6935T C7028T T7657C A8188G A8860G G9055A T9698C A9852T A10550G T11299C A11467G G11719A A12308G G12372A C14167T T14212C C14766T T14798C A15326G T16093C C16148T G16153A T16224C C16286T T16311C G16472C T16519C |
| NA18541 | A73G A263G 514delC 515delA A750G A1438G A2706G A4769G A6302G T6497C C7028T 8281delC 8282delC 8283delC 8284delC 8285delC 8286delT 8287delC 8288delT 8289delA T8380C A8860G G9329A G11150A G11719A C14766T C14953G A15326G A16182C A16183C T16189C T16217C C16261T |
| NA18542 | A73G T152C 248delA A263G T310N T489C A750G A1438G A2706G T3552A A4715G A4769G G5821A A6338G C7028T C7196A G7853A G8584A A8701G A8860G T9540C A9545G A10398G C10400T T10873C G11719A G11914A C12705T A13263G G13928A T14318C T14463C C14766T T14783C G15043A G15301A A15326G A15487T C16223T T16298C C16327T T16519C |
| NA18545 | A73G T204C 248delA A263G T310N 514delC 515delA A750G A1438G A2706G C3970T A4732G A4769G G5147A G6018A T6392C G6962A C7028T A8860G G10310A T10609C C10976T G11719A G12406A C12633T C12882T G13928C G14476A C14766T A15326G A16182N A16183C T16189C C16232A T16249C T16304C T16311C T16519C |
| NA18547 | A73G 248delA A263G T310C T489C A750G A1438G C1677T 2226insA A2706G T2887C T3552A T4696C A4715G A4769G G6026A C7028T C7196A G8584A A8701G A8860G T9540C A9545G C9752T T10304C A10398G C10400T T10873C G11719A G11914A G11969A T12624C A12672G C12705T T12954C A13263G T14318C C14766T T14783C G15043A T15204C G15301A A15326G A15487T C16223T T16298C T16311C C16327T T16357C T16519C |
| NA18550 | A73G C150T T159C T199C A263G T489C A750G A1438G A1643G A2706G G4048A C4071T A4164G A4769G T5004C A5351G G5460A C6455T T6680C C7028T T7684C G7853A T8404C A8701G A8860G G9305A T9540C T9824C C10159T A10398G C10400T T10873C G11719A T11944C C12405T C12705T T12811C C14766T T14783C G15043A G15301A A15326G T15479C T16092C C16192T C16223T T16297C T16311C |
| NA18552 | A73G C150T A263G 310insC G709A A750G T1119C A1438G A2706G C3435T C3497T C3571T A4769G G5758N G5821A C7028T C7990T 8281delC 8282delC 8283delC 8284delC 8285delC 8286delT 8287delC 8288delT 8289delA A8860G T9128C G11440A G11719A C14766T A14818G A15326G G15346A T16140C A16183C C16187N T16189C T16217C G16274A A16335G T16519C |
| NA18553 | A73G G185A A189G T195C A234G A263G 514delC 515delA A1438G A2706G C3543T A3861G A3873G A4562G A4769G A5581G C7028T G7853A A8860G G8994A C9248T C9532N T9861C G10373A T11318C G11719A T12136C C12705T G13194A A13434G G13928C C14158T C14392T C14766T C14953T C15211T A15326G C16111T T16172C A16183C T16189C C16223T T16311C T16362C T16519C |
| NA18555 | A73G G185A A189G A263G C309T T310C G709A A750G A1438G A2706G A4769G T5981C C7028T A7076G T8277C 8278insN T8279C A8860G T10031C A10398G C11061T G11719A A12950G T13215C A13269G A13681G C14766T A15326G T16092C G16145A A16182C A16183C T16189C T16311C G16390A T16519C |
| NA18558 | A73G C150T A263G 310insC T489C 514delC 515delA A750G C752T T1107C A2706G A4769G C4883T C5178A A5301G C7028T A8479G A8701G A8860G A9180G T9540C A10397G A10398G C10400T T10873C G11719A T11944C A12026G C12705T T14783C G15043A G15301A A15326G T16092C T16172C A16182N A16183N T16189C C16223T C16266T T16362C |
| NA18560 | A73G T195C 248delA A263G 514delC 515delA A750G T1005C A1438G T1824C A2706G T3278C C3970T A4769G A4811G T6392C C7028T A7828G A8860G G10310A T10535C G10586A G11719A T12338C G13708A G13928C C14766T A15218G A15326G T16092A C16291T T16304C |
| NA18561 | A73G 248delA A263G T310N 514delC 515delA A750G A1438G C1734T A2706G C3970T A4769G T5628C T6392C G6962A C7028T T7738C A8860G G10310A T10609C T10828C G11719A G12406A C12882T T13635C T13899C G13928C A15326G C15402T A16183C T16189C T16304C T16519C |
| NA18562 | A73G T146C A263G T310N T489C A750G A1438G A2706G G3010A A4769G C4883T C5178A T6680C C7028T C8414T A8701G A8860G T9540C A10398G C10400T T10873C G11719A C12705T C14668T C14766T T14783C G15043A G15301A A15326G C16223T C16294T T16362C |
| NA18563 | A73G C150T A263G A750G A1438G A2706G T2887C A4769G G5231A G5417A C7028T A8860G G11719A A12358G G12372N C12705T C14766T A15326G C16223T C16257A C16261T T16519C |
| NA18565 | A73G T152C A263G T489C A750G A1438G A2706G G3010A C3206T A4769G C4883T C5178A A5582G C7028T C8414T T8473C A8683G A8701G A8860G T9540C A10398G C10400T T10873C G11719A C12705T C14443T C14668T C14766T T14783C T14979C G15043A G15301A A15326G G15596A G16129A C16223T T16362C |
| NA18566 | A73G T195C 248delA A263G T489C 593insC A750G A1118T A1438G A2706G T3552A A4715G A4769G A6113G C7028T C7196A G8584A A8701G A8860G T9540C A9545G A10398G C10400T T10873C G11719A G11914A T12161C C12705T A13263G G13708A C14315T T14318C C14766T T14783C G15043A G15301A A15326G A15487T T16093C C16179A C16223T T16288C T16298C C16327T T16519C |
| NA18567 | A73G A193G A263G T310N 514delC 515delA G709A A750G A1438G A2706G A4769G T5465C T5999C T6293C C7028T 8281delC 8282delC 8283delC 8284delC 8285delC 8286delT 8287delC 8288delT 8289delA A8860G G9123A G11719A C14049T C14751T C14766T A15326G 16182delA 16183delA T16189C T16217C C16261T A16299G T16311C T16519C |
| NA18569 | A73G 248delA A263G 514delC 515delA C548T A750G A1438G C3970T C4086T A4769G A6098G T6392C G6962A C7028T A8860G G9053A G9548A C10211T G10310A T10609C G11719A G12406A C12882T G13759A G13928C G14198A A14683G C14766T A15326G A15534G G16129A A16162G T16172C T16304C T16519C |
| NA18570 | A73G A263G T489C A547G T593C G709A A750G A1438G A2706G A4769G A4833G T4973C T5108C C7028T C8161T T8200C A8701G A8860G T9540C G9966A A10398G C10400T T10873C G11719A C12705T G14569A C14766T T14783C G15043A G15301A G15323A A15326G G15497A C16223T T16362C T16519C |
| NA18571 | A73G T152C A235G 248delA A263G T310N A750G T1005C C1009T A1438G T1824C A2706G C3970T A4769G T6392C C7028T A7403G A7828G A8860G G10310A T10535C G10586A G11719A T12338C G13708A G13928C C14766T A15326G A16051G T16362C |
| NA18572 | A73G T152C A235G A263G 514delC 515delA A663G A750G A1438G A1736G A2706G G4113A T4248C A4769G A4824G A5514G C7028T C8794T A8860G T9126C G11719A C12705T C14766T G15217A A15326G C16223T C16290T G16319A T16362C G16390A |
| NA18573 | A73G C150T T152C G185A A263G C456T T489C 514delC 515delA T681C A750G C1048T T1107C A1438G A2706G A4769G C4883T A5153G C5178A A5301G T6253C C7028T A8701G A8860G A9180G T9540C A10397G A10398G C10400T T10873C G11719A A12666C C12705T C14766T T14783C G15043A T15287C G15301A A15326G T15470C T16092C C16148T A16183C T16189C C16223T T16362C T16519C |
| NA18574 | A73G T146C C151T T152C T199C A234G A263G 310insC T489C 514delC 515delA A750G A1438G A2706G C4071T A4769G C4850T T5442C C6455T C7028T G7337A A8701G A8860G T9540C T9824C T9957C A10398G C10400T T10861C T10873C C11665T G11719A T12091C G12561A C12705T G13590A C14766T T14783C G15043A G15301A A15326G C16071T C16223T C16295T T16519C |
| NA18576 | A73G T146C C150T A263G C456T T489C T681C A750G C1048T T1107C A1438G A2706G G4048A A4769G C4883T A5153G C5178A A5301G T6253C C7028T A8701G A8860G T8979C A9180G T9540C A10397G A10398G C10400T T10810C T10873C G11719A C12705T C14766T T14783C C14821T G15043A C15286T G15301A A15326G C15451T A15724G A16183C T16189C C16223T T16362C |
| NA18577 | A73G A263G T489C G709A A750G A1438G A2706G 3167insC C4140T A4769G G5471A C7028T A7250G A8701G T8793C G8856A A8860G T9540C G9932A T10245C A10398G C10400T G10646A T10873C G11719A T11732C C12549T C12705T G13135A A13152G T14502C C14766T T14783C C15040T G15043A T15071C T15109C A15218G G15301A A15326G T16086C G16129A T16172C T16189N C16223T T16311C |
| NA18579 | A73G T152C A235G A263G 310insC A663G A750G A1438G A1736G A2706G T4248C A4769G A4824G C7028T C8794T A8860G G11719A C12705T C14766T A15326G T16092C C16223T C16290T G16319A T16362C |
| NA18580 | A73G T195C A263G T489C A750G A1438G A2706G G3010A A4769G C4883T C4904T C5178A C7028T C8414T A8701G A8860G C9407T C9449T T9540C A10398G C10400T T10873N G11719A C12092A C12705T C14668T C14766T T14783C G15043A A15236G G15301A A15326G C16192T C16223T |
| NA18582 | A73G C150T T199C A263G T310N T489C A750G A1438G A2706G G4048A C4071T A4164G A4769G A5351G G5460A C6455T T6680C C7028T T7684C G7853A A8701G A8860G T9540C T9824C A10398G C10400T T10873C G11719A C12405T C12705T T12811C C14766T T14783C G15043A G15301A A15326G G16129A C16192T C16223T T16297C |
| NA18583 | A73G 248delA A263G A750G A761G T1005C A1438G T1824C A2706G C3970T A4769G A5978G C6156T T6392C G6570T C7028T A7828G G8152A A8860G G10310A T10535C G10586A G11719A T12338C G13708A A13722G G13928C A14133G C14766T A15326G C15418T C16067T C16192T T16271C T16304C T16311C |
| NA18591 | A73G T146C T152C A263G T489C 514delC 515delA A750G A1438G A2706G G3010A C3206T A4769G C4883T C5178A C7028T C8414T T8473C A8701G A8860G T9540C A10398G C10400T T10873C G11719A C12705T A13350G C14668T C14766T T14783C T14979C G15043A G15301A A15326G G16129A C16148N C16223T A16265G C16270T T16362C T16519C |
| NA18592 | A73G 248delA A263G 567insC 567insC 567insC 567insC A750G A1438G A2706G C3970T A4769G C5263T T6392C C6653T C7028T T7741C G8020A C8575T T8603C A8860G G9266A C9764T G10310A G11719A T12354C G12630A T12879C G13928C C14766T A15326G T15670C T16172C C16218T T16304C T16311N |
| NA18593 | A73G A263G 310insC T489C A750G A1041G A1438G A2706G T3394C G4491A A4769G C7028T A8701G A8860G A9242G T9540C A10398G C10400T T10873C G11719A G11914A G11963A C12705T T14308C C14766T T14783C G15043A G15301A A15326G C16223T C16234T A16316G T16362C |
| NA18598 | A73G 248delA A263G A750G T1005C A1438G T1824C A2706G C3970T A4769G T6392C C6566T C7028T A7828G A8860G G10310A T10535C G10586A G11719A T12338C G13708A G13928C C14766T G14831A A15326G G16129A A16166G T16189C A16203G T16304C T16519C |
| NA18603 | A73G T146C 248delA A263G T310N 514delC 515delA A750G A1438G C1734T A2124G A2706G C3970T A4769G T5628C T6392C G6962A C7028T T7738C A8860G G10310A T10609C G11719A G12406A C12882T G13135A G13928C C14766T A15326G C15402T A15924G T16189C A16284G T16304C |
| NA18605 | A73G T146C C150T C151T T152C A263G T489C A750G T1107C A1438G A2706G C3546T C4025T A4200T T4216C A4769G C4883T C5178A A5301G C7028T A8701G A8860G T9540C A10289G A10397G A10398G C10400T T10873C G11719A C12705T A13105G C14766T T14783C A14927G G15043A G15301A A15326G T15622C A16051G T16189C C16223T C16320T T16362C G16390A |
| NA18606 | A73G 248delA A263G 514delC 515delA C548T A750G A1438G A3565G C3970T C4086T A4769G T5277C A6098G T6392C G6962A C7028T G7269A A8860G G9053A G9548A C10211T G10310A T10609C G11719A G12406A C12882T G13759A G13928C C14766T A15326G G16129A A16162G T16172C T16304C T16519C |
| NA18608 | A73G A215G A263G T310N T318C A326G T489C A723C A750G T1095C G1289A A1438G A2706G A4769G A5319G C6531T C7028T G7642A A8108G A8701G A8860G A8906G T9540C T9950C A10398G C10400T T10873C G11719A G11969A C12705T A14029G T14502C C14766T T14783C G15043A G15301A A15326G C16169T C16173T C16223T T16243C T16519C |
| NA18609 | A73G 248delA A263G T310N A750G A761G T1005C A1438G T1824C A2706G C3970T A4769G T4823C C5318T A5978G C6156T T6392C G6570T C7028T A7828G G8152A A8860G G10310A T10535C G10586A A10978G G11719A T12338C A13470G G13708A A13722G G13928C A14133G C14766T A15326G C15418T C16067T C16234N T16271C T16304C |
| NA18611 | A73G A235G A263G 514delC 515delA A663G A750G 955insC T961C A1438G G1709A A1736G A2706G T3338C G3915A T4248C A4769G A4824G A6788G C7028T A8563G C8794T A8860G C11536T G11719A C12705T C14766T A15326G T16126C G16153A C16223T A16235G C16290T G16319A T16519C |
| NA18612 | A73G C150T A263G T310N A750G A1438G A2706G T2887C A3257G A4769G G5231A G5417A C7028T T7080C A8158G C8263T A8860G C10088T G11719A A12358G G12372A C12705T C14766T A15326G C16188T C16223T C16257A C16261T A16299G |
| NA18614 | A73G A263G 514delC 515delA A750G A1438G A2706G T4703C A4769G T5093C A6485G C7028T 8281delC 8282delC 8283delC 8284delC 8285delC 8286delT 8287delC 8288delT 8289delA A8860G T11254C G11719A A13269G G13368A C14766T A15326G G16129A 16182delA 16183delA T16189C C16261T |
| NA18615 | A73G T152C A235G A263G T310N 514delC 515delA A663G A750G A1438G A1736G A2706G G3010A T4248C A4769G A4824G C7028T C8794T A8860G T11465C G11719A C12705T C14766T A14965G A15326G C16223T A16230G C16290T G16319A T16362C |
| NA18616 | A73G A200G A263G T310N G316A A750G A827G A1438G A2706G A4769G C6287T C7028T 8281delC 8282delC 8283delC 8284delC 8285delC 8286delT 8287delC 8288delT 8289delA A8860G G11719A G11914A A13419G A13942G C14766T A15038G A15326G C15535T G15930A A16182C A16183C T16189C T16217C T16519C |
| NA18617 | A73G T146C A263G T310C G316A 514delC 515delA G709A A750G A1438G A2706G A4769G T5465C C7028T 8281delC 8282delC 8283delC 8284delC 8285delC 8286delT 8287delC 8288delT 8289delA A8860G G9123A T10238C C11632T G11719A C12237T C14766T A15038G A15326G A16182C A16183C T16189C T16217C C16261T T16519C |
| NA18618 | A73G T199C A263G T489C G513A 514delC 515delA A750G A1438G A2706G G3882A C4071T A4769G C4850T T5442C C6455T C7028T A8701G A8860G T9540C T9824C A10398G C10400T T10873C C11665T G11719A T12091C C12705T C14766T T14783C G15043A G15301A A15326G C16223T C16295T G16319A T16519C |
| NA18619 | A73G C151T A197G A263G A546G A750G A827G A1438G A2706G G3010A A4769G A6891G C7028T 8281delC 8282delC 8283delC 8284delC 8285delC 8286delT 8287delC 8288delT 8289delA A8860G A9120G G11719A G11914A C12135T A13942G C14766T A15326G C15535T G15930A 16183delA C16186T T16189C T16217C C16234T T16519C |
| NA18620 | A73G 248delA A263G 514delC 515delA C548T A750G A1438G A2706G C3970T C4086T A4769G T6392C G6962A C7028T A8860G G9053A G9548A T10208C C10211T G10310A T10609C A11653G G11719A G12406A C12882T G13759A G13928C C14766T C15145T A15326G G16129A A16162G T16172C T16304C T16519C |
| NA18621 | A73G T152C T199C A235G A263G 514delC 515delA A663G A750G A1438G A1736G A2706G T4248C A4769G A4824G T4977C G5460A C7028T A7325G C8794T A8860G G11719A C12705T C14766T A15326G C15436T C16223T C16290T T16311C G16319A T16362C T16519C |
| NA18622 | A73G T152C 248delA A263G T310N T489C G709A A750G A1438G A2706G A4715G A4769G A6752G C7028T C7196A G8584A A8701G A8860G T9090C A9494G T9540C T10208C A10398G C10400T T10873C G11719A C11782T C12705T C14766T T14783C G15043A G15301A A15326G A15487T T15784C C16185T C16223T C16260T T16298C T16311C T16519C |
| NA18623 | A73G C150T A263G T489C 514delC 515delA A750G C752T T1107C A2706G A4769G C4883T C5178A A5301G T6112C C7028T G7757A A8701G T8793C A8860G A9180G T9540C A10397G A10398G C10400T T10873C G11719A T11944C A12026G C12705T C14766T T14783C G15043A G15301A A15326G A16164G A16182N A16183N T16189C C16223T C16266T T16362C |
| NA18624 | A73G 248delA A263G 514delC 515delA A750G T1005C A1438G T1824C A2706G C3970T A4769G A4811G T6392C C7028T A7828G G8697A A8860G G10310A T10535C G10586A G11719A T12338C G13708A G13928C C14766T C15211T A15326G T16092A C16291T A16299G T16304C |
| NA18625 | A73G C194T A263G 310insC T489C 514delC 515delA A750G A1382C A1438G A1978G A2706G G3010A A4769G C4883T C5178A C7028T G8020A C8414T G8485A A8701G A8860G C8964T C9296T T9540C T9824A A10398G C10400T T10873C G11719A C12705T A12950G T13182C C14668T C14766T T14783C G15043A G15301A A15326G C15832T T16093C C16223T T16362C T16519C |
| NA18626 | A73G T152C 248delA A263G T310N A750G T1005C A1438G T1824C A2706G A3547G C3970T A4769G T6392C C7028T A7828G A8860G T9128C G10310A T10535C G10586A T11485C G11719A T12338C G13708A G13928C A14687G C14766T A15326G G15884A A16203G C16291T T16304C T16311C A16335G T16352C T16519C |
| NA18627 | A73G G207A A263G G499A A750G A827G A1438G G1719A A2220G A2706G G2831A A4769G G4820A G6023A T6216C T6413C C7028T T7993C 8281delC 8282delC 8283delC 8284delC 8285delC 8286delT 8287delC 8288delT 8289delA A8860G G11719A G13590A C14766T G15301A A15326G C15535T T16136C A16183C T16189C T16217C A16309G C16354T T16519C |
| NA18628 | A73G C150T A263G A750G A1438G G1888A A2706G A4769G G5231A G5417A G6026A C7028T G8251A T8404C A8860G T9581C G11719A A12358G G12372A C12705T C14766T A15326G T16189C C16223T C16257A C16261T |
| NA18629 | A73G 248delA A263G A750G T1005C A1438G T1824C A2281G A2706G G3010A C3970T A4769G T5493C T6392C C7028T A7828G A8860G T10265C G10310A T10535C G10586A G11719A T12338C G13708A G13928C C14766T A14769G A15010G A15326G T16304G |
| NA18630 | A16T A73G C150T A263G T489C 514delC 515delA A750G C752T T1107C G1719A A2706G A4769G C4883T C5178A A5301G C7028T 7508insT T8516C A8701G A8860G A9180G T9540C A10397G A10398G C10400T T10873C G11719A T11944C A12026G C12705T T14308C C14766T T14783C G15043A G15301A A15326G A16164G T16172C A16182N A16183C T16189C C16223T C16266T T16362C |
| NA18631 | A73G T152C 248delA A263G T489C A750G A1438G A2706G T3552A A4715G A4769G A5978G G6026A C7028T A7100G C7196A G8584A A8701G A8860G T9540C A9545G T10248C A10398G C10400T T10873C G11719A G11914A G11969A C12705T A12780G A13263G T13581C T14318C C14766T T14783C G15043A T15204C A15236G G15301A A15326G A15487T T16093C C16223T T16298C C16327T T16519C |
| NA18632 | A73G T152C A235G 514delC 515delA A663G A750G A1438G A1736G A2706G G3531A G3849A T4248C A4769G A4824G C7028T G7316A A8459G A8659G C8794T A8860G G11719A C12705T C14067T C14766T G15314A A15326G C16223T C16290T G16319A T16362C |
| NA18633 | A73G T195C A263G T489C A750G A1438G A2706G G3010A A4769G C4883T C4904T C5178A C7028T C8414T A8701G A8860G C9407T T9540C A10398G C10400T T10873C G11719A C12092A C12705T C14668T C14766T T14783C G15043A A15236G G15301A A15326G C16192T C16223T |
| NA18634 | A73G A263G T489C G709A A750G A1438G A2706G T3278C A4769G A4828N A4833G T5108C C7028T G7521A T8200C G8572A A8701G A8860G G9300A T9540C A10398G C10400T T10873C T11253C G11719A C12705T G14569A C14766T T14783C G15043A G15301A G15323A A15326G G15497A C16223T T16362C T16519C |
| NA18635 | A73G A263G T489C G709A A750G A1438G A2706G A4769G A4833G T5108C C5601T C7028T G7600A A8701G A8860G A9377G T9540C G9575A A9659G A10398G C10400T T10873C G11719A C12705T A13563G A13884G T14200C C14443T G14569A C14766T T14783C G15043A G15301A A15326G A16051G C16114T C16223T A16227G C16278T C16291T T16362C |
| NA18636 | A73G C150T T199C A263G T489C 567insC 567insC A750G A1438G A2706G G4048A C4071T G4092A A4164G A4769G A5351G G5460A C6455T T6680C C7028T T7684C G7853A A8701G A8860G T9540C T9824C A10398G C10400T T10873C G11719A C12405T C12705T T12811C C14766T T14783C G15043A G15301A A15326G A15679G G16129A T16189C C16223T T16297C |
| NA18637 | A73G T489C G709A A750G A1438G G2120A A2706G T3593C A4769G A4833G G4853A T5108C C5601T C7028T A8701G A8860G T8877C T9540C A10398G C10400T T10873C C11151T G11719A A12076G C12705T A13563G G14569A C14766T T14783C G15043A G15301A A15326G T16172C T16209C C16223T T16362C |
| NA18638 | A73G C150T T199C T204C A263G T489C A750G A1438G A2706G G4048A C4071T A4164G A4769G A5351G G5460A C6455T T6680C C7028T T7684C G7853A A8701G A8860G T9540C T9824C T10345C A10398G C10400T T10873C G11719A C12405T C12705T T12811C C14766T T14783C G15043A G15301A A15326G G16129A A16183C T16189C C16223T T16297C T16298C T16325C |
| NA18639 | A73G T146C T199C A263G T489C 514delC 515delA A750G A1438G A2706G C4071T A4769N C4850T C5194T T5442C C6455T C7028T T7692C A8701G A8860G T9540C T9708C T9824C T9957C A10398G C10400T T10873C C11665T G11719A T12091C G12561A C12705T G13590A C14766T T14783C G15043A G15217A G15301A A15326G T16172C C16223T C16295T T16519C |
| NA18640 | A73G A263G T310N T489C 514delC 515delA A750G C752T T1107C C1310T A2706G A4769G C4883T C5178A A5301G C6617T C7028T A7364N C7365N C7366N C7462N A8701G A8860N A9180G T9540C A10397G A10398G C10400T T10873C G11719A T11944C A12026G C12705T A13278G C14766T T14783C G15043A G15301A A15326G A15924G T16092C A16164G T16189C C16223T C16266T T16362C |
| NA18641 | A73G T146C 248delA A263G T319N 514delC 515delA A750G C1146N A1438G C1734T A2706G T3578N C3970T A4769N T5628C T6392C G6962A C7028T T7738C C8263N C8264N T8265N A8266N T8267N A8268N G8269N C8270N A8271N C8840N C8841N A8842N T8843N C8844N C8845N C8846N C8847N T8848N T8849N A8850N T8851N G8852N A8853N G8854N C8855N G8856N C10192N G10310A T10609C G11719A G12406A T12696C C12882T G13928C A14510N C14511N T14512N A14513N T14514N T14515N A14516N A14517N A14518N C14519N C14520N C14521N A14522N T14523N A14524N T14525N A14526N C14766T A15326G C15402T T16189C T16304C T16519C T16568N G16569N |
| NA18642 | A73G T152C A235G A263G A663N A750G A1438G A1736G A2706G T4248C A4769N A4824G C7028T C8794T A8860G G11719A C12705T C14766T T14788C A15326G C16223T C16290T G16319A T16362C |
| NA18643 | A73G G103A T131C T146C T199C T204C A263G T310N 514delC 515delA G709A A750G A1438G G1598A C1904N A2706G T3398C A4769G A4895G T5495N A5496N T5497N A5498N T5964C C7028T 8281delC 8282delC 8283delC 8284delC 8285delC 8286delT 8287delC 8288delT 8289delA G8584A C8829T A8860N T9950C T10173N A10398G G11719A A12361G G12610N A12612N C14766T C15223T A15326G C15508T A15662G T15850C A15851G G15927A C16111T T16140C A16183C T16189C C16234T T16243C T16311C G16390A T16519C |
| NA18644 | A73G C150T T195C T199C T217C A263G T489C A750G A1438G A2706G T3368C G4048A C4071T A4164G A4769G G5252A A5351G G5460A C6455T T6680C A6929G C7028T G7598A T7684C G7853A A8701G A8860G T9540C T9824C A10398G C10400T T10873C G11719A G12007A C12016T C12405T C12705T T12811C C14766T T14783C G15043A G15301A A15326G G16129A C16223T T16297C T16519C |
| NA18645 | A73G A235G 248delA A263G A750G T1005C C1009N A1438G T1824C A2706G C3970T A4769N T6392C C7028T C7492T A7828G A8860G C9099T G10310A T10535C G10586A G11719A T12338C G13708A G13928C C14766T A15326G T16311C |
| NA18646 | A73G A263G T489C A750G A1438G G2185A A2706G A4715G A4769G C5100T G6179A C7028T C7196A G8584A C8684T A8701G A8860G T9540C A10398G C10400T T10873C G11719A C12216T C12705T T14470C C14766T T14783C G15043A G15301A A15326G A15487T T16126C C16184T C16223T T16298C G16319A |
| NA18647 | A73G T152C C194T A263G T489C 514delC 515delA A750G A1382C A1438G A2706G G3010A A4769G C4883T C5178A A5674G C7028T G8020A C8414T A8577G A8701G A8860G C8964T C9296T T9540C T9824A A10398G C10400T T10873C G11719A A12358G C12705T C14668T C14766T T14783C G15043A G15301A A15326G T16172C T16362C T16519C |
| NA18648 | A73G C151T T152C A263G T489C T650C A750G A1438G A2706G G3010A C3206T A4769G C4883T C5178A 5894insC T6524C C7028T C8414T T8473C A8701G A8860G T9540C A10398G C10400T T10873C G11719A C12705T C13650G T14494N G14560A C14668T C14766T T14783C T14979C G15043A G15301A A15326G G16129A C16223T T16362C T16519C |
| NA18649 | A73G A200G A263G T489C 567insN G709A A750G A1438G A2706G 3167insC G3882A C4140T A4769G C7028T A7250G A8701G T8793C G8856A A8860G T9540C A10398G C10400T G10646A T10873C G11719A C12549T C12705T A13152G T14502C G14569A C14766T T14783C C15040T G15043A T15071C G15301A A15326G A16066G C16223T T16311C T16519C |
| NA18669 | A73G 248delA A263G T489C A750G A1438G A2706G T3552A A4715G A4769G G5821A A6338G C7028T C7196A G7853A G8584A A8701G A8860G T9540C A9545G A10398G C10400T T10873C G11719A G11914A A12634G C12705T A12810G A12909G A13263G T13635G T14318C T14470C C14766T T14783C G15043A G15301A A15326G A15487T T16189C C16223T T16298C C16327T T16519C |
| NA18674 | A73G T146C T199C A263G T489C 514delC 515delA A750G A1438G A2706G A3606G C4071T A4769G G4841A C4850T T5442C G6260A C6455T T6681C C7028T A8701G A8860G T9540C T9824C A10398G C10400T T10873C C11665T G11719A A11908G T12091C C12705T A13563G C14766T T14783C G15043A A15236G G15301A A15326G C16223T C16295T T16362C T16519C |
| NA18675 | C64T A73G A263G T346C G499N A750G A827G A1438G A2706G A3547G A4769G G4820A T4977C G6383A C6473T C7028T A8860G A9007G A9097G T9950C C11177T G11719A G13590A C14766T A15326G C15535T C16111T 16183delA T16189C T16217N G16483A T16519C |
| NA18682 | G1N A2N A73G T125N T127N C128N A263G T310N T318C T489C G513N 514delC A750G A1438G A2706G C4170T A4769G T5580N C7028T T8654C A8701G A8860N C9490N T9540C A10398G C10400T T10873C G11719A A12030G A12358G G12372A C12705T G14569A T14727C C14766T T14783C A15010G G15043A G15301A A15326G A15463G C15651T T16209C C16223T C16234T C16287T C16290T T16362C |
| NA18685 | A73G G94A A263G 310insC T489C A517G A750G A1438G A2706G G3010A G3316A A4769G C4883T C5178A T5964C C7028T T8119C C8414T A8701G A8860G C9536T T9540C A10398G C10400T T10873C C11215T G11719A C12705T T14470C C14668T C14766T T14783C C14860T G15043A G15301A A15326G A15924G T16092C C16223T T16362C |
| NA18689 | A73G G207A A263G T310N T408A G499A A750G A827G A1438G A2706G G3010A A4769G G4820A T6413C C7028T 8281delC 8282delC 8283delC 8284delC 8285delC 8286delT 8287delC 8288delT 8289delA A8860G G9055A A9338T T9615C G11719A G13590A C14766T A15326G C15535T T16136C A16183C T16189C T16217C T16519C |
| NA18691 | A73G T146C T152C 248delA A263G A281G 514delC 515delA A750G A1438G A2706G T3290C C3970T T4703C A4769G G5231A C5263T T6392C C7028T G8839A A8860G G10310A T10915C G11719A T11776C A12612G G12630A G13928C G14016A C14766T A15326G T15670C T15908C A16207G T16304C A16399G |
| NA18692 | A73G A263G T310C T489C A750G A1438G A2706G G3010A A4769G C4883T C5178N C7028T C8414T A8701G A8860N C9536T T9540C A10398G C10400T C10556N T10873C C11215T G11719A C12705T G13135A C14668T C14766T T14783C T14857C G15043A G15301A A15326G C16223T T16362C |
| NA18694 | G1N A2N A73G A263G 310insC C338N T489C A750G A1438G A2706G G3010A A4769G C4883T C5178A C7028T C8414T A8701G A8860G G9192A T9540C A10398G C10400T T10873C G11696A G11719A C12705T G13711A C14668T C14766T T14783C G15043A G15301A A15326G C16184T C16223T T16311C T16362C |
| NA18696 | G1N A2N A73G A263G T310N C431T T489C 514delC 515delA A750G A1438G A2706G G3010A A4769G C4883T C5178A C6689T C7028T G8020A C8414T C8532T A8701G A8860N C8861N A8862N A9150G T9540C A9894G C10181T A10398G C10400T T10873C G11719A C12705T C14668T C14766T T14783C G15043A G15301A A15326G T15440C G15764N A15951G T16172C C16223T C16287T G16319A T16362C |
| NA18697 | A73G C151T T152C A200G A235G A263G 514delC 515delA A663G A735G A750G A1438G A1736G A2706G T4248C A4769G A4824G C7028T C8794T A8860G C10864T G11719A C12705T C14766T A15326G T16136C C16223T C16290T G16319A T16362C A16463G |
| NA18699 | A73G A263G T489C 567insN G709A A750G A1438G A2706G 3167insC A3397G C4140T A4769G C7028T A7250G A8701G T8793C T8843N G8856N A8860N C8861N A8862N T9540C G9932A T10245C A10398G C10400T G10646A T10873C G11719A C12549T C12705T G13135A A13152G T14502C C14766T T14783C C15040T G15043A T15071C T15109C A15218G G15301A A15326G G16129A C16223T T16311C G16569N |
| NA18702 | A73G C194T A263G 310insC T489C 514delC 515delA A750G A1382C A1438G A2706G G3010A A3144G A4769G C4883T C5178A C7028T G8020A C8414T A8701G A8860G C8964T C9296T T9540C G9804A T9824A A10398G C10400T T10873C G11719A C12705T C14668T C14766T T14783C G15043A G15301A A15326G A15422G C16223T T16362C T16519C |
| NA18704 | A73G G103A T152C T195C T204C A263G T310N 514delC 515delA G709A A750G A1438G G1598A A2706G G3834A A4769G G4924A C7028T G8584A C8829T A8860G T9950C A9980G A10398G G11719A A12361G A14028G G14410A C14766T G14831A C15223T A15326G C15508T A15662G A15851G G15927A T16140C A16166G 16183delA T16189N T16243C T16519C |
| NA18707 | A73G T146A T199C A263G T489C 514delC 515delA A750G A1438G A2706G A3606G C4071T A4769G C4850T T5442C C6455T C7028T C7160T A8701G A8860N T9540C T9824C A10398G C10400T T10873C C11665T G11719A T12091C C12705T A13203G C14766N T14783N G15043A A15236G G15301A A15326G T16519C |
| NA18708 | A73G T152C 248delA A263G A750G A1438G A2706G T3396C C3970T C4086T T4733C A4769G T6392C G6962A C7028T A8860G G9053A G9548A G10310A T10609C G11719A G12406A C12882T G13759A G13928C C14766T A15326G A15373G G16129A T16172C T16304C T16519C |
| NA18739 | A73G C194T A263G 310delT T489C 514delC 515delA A750G A1382C A1438G A2706G G3010A A4769G C4883T C5178A C7028T G8020A C8414T A8701G A8860G C8964T C9296T T9540C T9824A A10398G C10400T T10873C G11719A A12358G C12705T G13707A C14668T C14766T T14783C G15043A G15301A A15326G A16183N T16189C C16223T T16356C T16362C T16519C |
| NA18740 | A73G C150T A263G 310insC A750G A1438G A2052N T2053N T2054N A2706G T3338C A4129G A4769G G5231A G5417A C7028T A8860G G11719A T12354C A12358G G12372A A12612G C12705T C14766T A15326G G16129A C16223T C16248T C16257A C16261T |
| NA18741 | A73G C150T A263G A750G A1438G A2706G A4769G G5147A G5231A G5417A C7028T G7805A A8860G G9755A A9821G A10328T G11719A A11968T A12358G G12372A C12705T G12771A C14766T A15326G C16223T C16257A T16519C |
| NA18742 | A73G A263G T489C G709A A750G A1438G A2706G T3593C A4769G A4833G T5108C C5601T A6932G C7028T T7705C A8701G A8860G T8877C G8896A T9540C A10398G C10400T T10873C A11002G G11719A C12705T A13563G T13926C G14569A C14766T T14783C G15043A G15301A A15326G C16223T T16362C T16519C |
| NA18743 | A263G T480C A750G A1438G A2706G G4655A A4769G C6569T C7028T A8860G T14110C T15115C A15326G T16311C |
| NA18744 | A73G A263G G499A A750N A827G A1438G A2706G A4769G G4820A G6962A C7028T A7202G G7521A 8281delC 8282delC 8283delC 8284delC 8285delC 8286delT 8287delC 8288delT 8289delA A8860G T9101G A11239G G11719A G11914A G13590A A14587G C14766T A15326G C15535T T16136C A16175G A16182N A16183C T16189C T16217C C16218T T16519C |
| NA18745 | A73G A263G A302N T489C A503G A750G A1438G A2706G C2835T A4715G A4769G G6179A C7028T C7196A C7958T G8584A C8684T A8701G A8860G T9540C A10398G C10400T T10873C G11176A G11719A C12705T T14470C C14766T T14783C G15043A G15301A A15326G A15487T C16184T T16189C C16223T T16298C G16319A G16438A G16470A G16471A G16473A |
| NA18747 | A73G C150N T152N A263G A750G A1438G G1664A A2706G A4769N G5231A G5417A C7028T A8860G A9156G T9230C G11719A A12358G G12372A C12705T C14766T A15326G T16092C G16145A T16172C C16223T C16245T C16257A C16261T T16519C |
| NA18748 | A73G C150T A263G 513insC 513insA 513insC 513insA A750G A1166N A1167N A1168N G1169N G1170N A1171N C1172N C1173N T1174N A1438G G1664A A2706G A4769G T5075N T5077N T5078N A5079N G5231A G5417A C7028T C7399N C7400N C7401N C7402N A7403N C7404N C7405N C7406N T7407N A7408N C7409N C7410N A7411N C7412N A7413N C7414N A7415N T7416N T7417N C7418N G7419N A7420N A7421N G7422N A7423N A7424N A9156G A10147N G11719A A12358G G12372A C12705T C14766T A15326G T16092C G16145A T16172C C16223T C16245T C16257A C16261T |
| NA18749 | A73G T152C A263G T489C 567insC 567insC 567insC G709A A750G A1438G A2706G A4035G C4140T A4769N C7028T A7250G A8701G T8793C G8856A A8860G T9540C A10398G C10400T G10646A T10873C G11719A C12549T C12705T A13152G T13602C T14502C A14755G C14766T T14783C C15040T G15043A T15071C A15218G G15301A A15326G A16066G T16086C C16223T T16311C |
| NA18753 | A73G 248delA A263G T489N A750G A1438G A2706G A4715G A4769G G5460A A6752G C7028T C7196A G8584A A8701G G8838A A8860G T9090C T9540C T10208C A10398G C10400T T10873C G11719A C12705T A13105G A13434G C14766T T14783C G15043A G15301A A15326G A15487N T15784C C16185N C16223T C16260T T16298C |
| NA18755 | A73G T146C T152C T195C C198T T199C A263G 310insC T489C A750G A929T A1438G A2706G G3277A C4071T T4216C A4769G C4850T T5442C C6455T C7028T A8701G A8860G T8939N C8940N T9540C T9824N A10398G C10400T T10873C A10921G C11665T G11719A T12091C C12705T C14684T C14766T T14783C G15043A G15301A A15326G A15363G C16192T C16223T T16224C C16295T T16519C |
| NA18756 | A73G T146C T199C A263G T489C 514delC 515delA A750G A1438G A2706G G3882A C4071T A4769G C4850T T5442C C6455T C7028T C8574T A8701G A8860G T9540C T9797C T9824C A10398G C10400T T10873N C11665T G11719A C11815A T12091C C12187T C12705T C12906A C14766T T14783C G15043A G15301A A15326G C16223T C16295T T16362C T16519C |
| NA18757 | A73G C150T A263G C456T T489C 514delC 515delA A750G C752T T1107C A2706G A4769N C4883T C5178A A5301G C7028T A8701G A8860G A9180G T9540C C10399T T10873C G11150A G11719A T11944C A12026G C12705T T13926C C14766T T14783C G15043A G15301A A15326G A16164G T16172C 16189delT C16223T C16266T T16362C |
| NA18758 | A73G T204C G207A A263G 514delC 515delA G709A A750G A1438G G1598A A2706G T4418C A4769G C7028T 8281delC 8282delC 8283delC 8284delC 8285delC 8286delT 8287delC 8288delT 8289delA G8584A A8784G C8829T A8860G T9950C A10398G G11518A G11719A A12361G C12585T G14384A C14766T G15172A C15223T A15326G C15508T A15851G G15927A T16140C A16183C T16189C T16243C T16519C |
| NA18759 | A73G G207A 248delA A263G G709A A750G A1438G A2045G A2706G A3434G C3970T A4769G A5474G G5585A A5894G G5913A A5978G T6392C C7028T A8860G T9854C G10310A G10320A A11065G T11485C G11719A C12621T G13928C C14766T T14783C T14971C A15326G T16093C C16260T T16298C C16355T T16362C |
| NA18760 | A73G A93G T146C T489C 514delC 515delA 567insC 567insC 567insC 567insC G709A A750G A1438G A2706G 3167insC C4140T A4769G C7028T A7250G A8701G T8793C G8856A A8860G T9540C A10398G C10400T G10646A T10873C G11719A C12549T C12705T G13135A A13152G T14502C A14564G C14766T T14783C C15040T G15043A T15071C A15218G G15301A G15323A A15326G G16129A C16193T C16223T T16311C T16357C A16497G |
| NA18761 | A73G 248delA A263G 514delC 515delA A750G A1438G A2706G C3970T A4715G A4769G T6392C T6515C G6962A C7028T A8860G T9021C G9053A G10310A T10609C G11719A G12406A A12418a G12771A C12882T G13759A G13928C C14766T A15326G G16129A T16172C T16304C T16519C |
| NA18763 | A73G G185A A189G A263G G709A A750G A1438G A2706G A4769G C5263T C7028T T8277C 8278insN T8279C T8654C A8860G A9210G T10031C G10373A A10398G C11061T G11176A G11719A A12950G A13681G C14163T C14766T A15218G A15326G T16172C A16182N A16183C T16189C T16311C T16519C |
| NA18764 | A73G A263G T489C G709A A750G A1438G A2706G A4769G A4833G T5108C A5378G C7028T C7867T T8200C A8701G A8860G T9540C A10398G C10400T T10873C G11719A G11887A A12040G C12705T G14569A C14766T T14783C G14905A G15043A G15301A G15323A A15326G G15497A C16184N C16223T C16290T T16362C T16519C |
| NA18765 | A73G A263G A291N T489C 514delC 515delA A750G A1438G A2706G G3010A T3336C T3644C A4769G C4883T T5048C C5178A G5262A C7028T G8152A G8387A C8414T A8701G A8860G T9540C A10042G A10398G C10400T T10873C G11719A C12705T C14668T C14766T T14783C G15043A G15301A A15326G C16167T T16172C C16174T C16223T C16287T T16362C |
| NA18767 | A73G T146C C150T A263G T391C C456N T489C T681C A750G C1048T T1107C A1438G A2706G G4048A A4769G C4883T A5153G C5178A A5301G T6253C C7028T A8701G A8860G A9180G T9316C T9540C A10397G A10398G C10400T T10810C T10873C G11719A C12705T C14766T T14783C G15043A G15301A A15326G A15496G A15724G A16183N T16189N C16223T T16362C |
| NA18768 | A73G A263G T489C A750G A1438G A2706G G3010A A4769G C4883T C5178A G5262A T5628C C7028T G7419A T7581C T7783C C7819N C8414T A8701G A8860G T9540C A10398G C10400T T10873C G11696A G11719A T12130C A12358G C12705T C12717T C14668T C14766T T14783C G15043A C15295T G15301A A15326G T16086C C16223T T16362C |
| NA18769 | A73G C150T T199C A263G T489C A750G A1438G T1694C A2706G G4048A C4071T A4164G T4502C A4769G A5351G G5460A C6455T T6680C C7028T A7375G T7684C G7853A A8701G A8860G T9540C T9824C A10398G C10400T T10873C C11659T G11719A C12405T C12705T T12811C C14766T T14783C G15043A G15301A A15326G T15618C G16129A T16189C C16223T T16297C A16300G T16519C |
| NA18770 | A73G A263G 514delC 515delA A750G A1438G A2706G A3221G G3483A A4769G G5237A C7028T G7789A 8281delC 8282delC 8283delC 8284delC 8285delC 8286delT 8287delC 8288delT 8289delA A8860G C9968T C11207T T11353C G11719A C13547T C14766T A15326G G16153A A16181N A16182N A16183C T16189C G16213A T16217C C16261T C16292T T16362N T16519C |
| NA18771 | A73G C150T C182T T199C A263G 456delC T489C A750G A1438G T1806C A2706G G4048A C4071T A4164G A4769G A5351G G5460A C6455T T6680C C7028T T7684C G7853A A8701G A8860G T9540C T9824C A10398G C10400T C10728T T10873N G11719A C12405T C12705T T12811C C14766T T14783C G15043A G15301A A15326G G16129A C16192T C16223T T16297C |
| NA18773 | A73G T152C G207A A235G 514delC 515delA A663G A750G A1438G A1736G A2706G T4248C A4769G A4824G C7028T A8459G C8794T A8860G A11084G G11719A C12705T C13943T C14067T C14766T A15326G C16223T C16290T G16319A T16362C |
| NA18778 | A73G C150T A263G G709A A750G T1119C A1438G A2706G C3435T C3497T C3571T A4769G C7028T 8281delC 8282delC 8283delC 8284delC 8285delC 8286delT 8287delC 8288delT 8289delA A8860G T9128C G9575A T10493C G11440A G11719A C14766T A15326G G15346A T16136C T16140C A16183N T16189N T16217C T16249C G16274A C16291T A16335G T16519C |
| NA18779 | A73G A263G A750G A1438G T1541C C2322A A2706G C3970T A4769G C7028T A8860G G11719A A12068G T12714C G13928C C14766T A15326G T16124C C16148T T16304C A16309G G16390A T16519C |
| NA18781 | A73G T152C A263G 514delC 515delA A750G A1438G T1541C A2706G C3204T G3316A A3828G C3970T A4769G T6815C C7028T A8860G T10101C G11719A T12714C G13928C C14766T A15326G T16189N C16192N T16304C A16309G G16390A T16519C |
| NA18785 | A73G A210G A263G 514delC 515delA T593C G709A A750N A1438G A2706G A3537G A4769G G5237A G5471A T5964C C6960T C7028T 8281delC 8282delC 8283delC 8284delC 8285delC 8286delT 8287delC 8288delT 8289delA G8584A A8860G T9950C G10325A A10398G A10523G G11719A C14766T A15235G A15326G T16140C A16183N T16189C C16262T C16266A T16519C |
| NA18789 | A73G T152C A263G T489C A750G A1438G A2706G G3010A A4769G C4883T A5097G C5178A A6578G C7028T G8020A C8414T A8701G A8860G T9540C A10049G C10181T A10398G C10400T T10873C T11116C G11719A T11992C C12705T C14668T C14766T T14783C G15043A G15301A A15326G C15370T T15440C A15951G A16158G C16223T A16284G C16287T A16293G G16319A |
| NA18790 | A73G 248delA A263G 514delC 515delA A750G T1005C A1438G T1824C A2706G C3970T A4769G A4811G T6392C C6935T C7028T A7828G A8860G G10310A T10535C G10586A G11719A T12338C G13708A G13928C C14766T A15326G T16092A C16291T T16304C |
| NA18791 | A73G T152C A235G A263G T449C 514delC 515delA A663G A750G A1438G A1736G A2706G T4248C A4769G A4824G T5162C C7028T C8794T A8860G G8921A C9440T A9524G T11087C G11719A C12705T C14766T G15172A A15326G C16223T C16290T G16319A T16362C |
| NA18794 | A73G A193G A263G 514delC 515delA A750G A1438G A2706G G3915A A4769G T5465C C7028T T8143C C8194T 8281delC 8282delC 8283delC 8284delC 8285delC 8286delT 8287delC 8288delT 8289delA T8715C A8860G G9123A G11719A G14323A C14751T C14766T A15326G A16182N A16183C T16189C T16217C C16261T A16299G G16390A T16519C |
| NA18939 | A73G A235G A263G 514delC 515delA A663N A750G A1438G A1736G 2150insA A2706G T4248C G4655A A4769N A4824G C7028T A8563G C8794T A8860G G10325A C11536T C11647T G11719A C12705T C14766T A15326G C16187T C16223T C16290T |
| NA18940 | A73G A263G T310N T489C 514delC 515delA A750G A1438G T2626C A2706G C2772T T4386C A4769G A4958G 5894insC C6455T C7028T A8701G A8860G A9299G T9540C T9824C A10398G C10400T T10873C T11017C A11084G G11719A T11722C C12705T G12771A G14364A C14766T T14783C G15043A G15301A A15326G T16209C C16223T T16324C |
| NA18941 | A73G T204C A263G A750G A1438G A2706G A4769G G5147A G5417A C7028T C7849T A8860G A9410G C10607T G11016A G11719A G12501A A13183G C13755T C14766T A14893G A15326G T16092C A16182C A16183C T16189C C16223T A16318G G16319A T16368C T16519C |
| NA18942 | A73G T152C A200G 248delA A263G T489C A750G A1438G A2706G A3520G A4715G A4769G T5492C A5894G A6752G C7028T C7196A T7270C G8584A T8654C A8701G A8860G T9090C T9540C A10398G C10400T T10873C C12705T C14766T T14783C G15043A G15301A A15326G A15475G A15487T T15784C 15940delT C16185T 16189delT C16223T C16260T C16294T T16298C A16302G G16526A |
| NA18943 | A73G C150T T199C T204C A263G C456T T489C A750G A1438G A2706G G4048A C4071T A4164G A4769G A5351G G5460A C6455T T6680C C7028T T7684C G7853A A8701G A8860G T9540C T9824C A10398G C10400T T10873C G11719A C12405T C12705T T12811C C14766T T14783C A14978G G15043A G15301A A15326G G16129A T16189C C16223T T16297C |
| NA18944 | A73G A263G 310insC T489C A750G A1438G A2706G G3010A A4769G C4883T C5178A C7028T G7702A T7783C C8414T A8701G A8860G T9540C G9804A A10398G C10400T T10873C C11215T G11719A C12705T C14668T C14766T T14783C G15043A G15301A A15326G A15874G C16223T T16304C T16362C |
| NA18946 | A73G A263G C431T T489C A750G A1438G A2706G G3010A T4859C C4883T C5178A C5743N G5744N G5745N G5746N A5747N A5748N A5749N A5750N A5751N A5752N G5753N G5754N C5755N C6410N C6689T C7028T G8020A C8414T A8701G A8860G T9540C T10084C C10181T C10223T A10398G C10400T T10873C G11719A A12172G C12705T C13239N C14668T C14766T T14783C G15043A G15301A A15326G T15440N A15951G C16223T C16287T G16319A T16362C A16399G |
| NA18947 | A73G 248delA A263G T310N 514delC 515delA A750G A1438G A2706G T3027C C3519T C3970T A4769G T5587C T6392C G6962A C7028T A8860G T10007C G10310A T10609C G11719A G12406A G12618A C12882T T13260C C13749T G13928C C14766T C15022T G15024A A15326G A15496G A16182N A16183C T16189C T16304C C16355T T16519C |
| NA18948 | A73G C150T A263G T310N 514delC 515delA A750G A1438G A2706G A4769G T5004C T5465C C7028T 8281delC 8282delC 8283delC 8284delC 8285delC 8286delT 8287delC 8288delT 8289delA A8860G G9123A G9932A T10238C T10915C G11719A T13143C C14766T A15326G A16182N A16183C T16189C T16217C C16261T C16268T T16288C T16311C T16519C |
| NA18949 | A73G T146C T199C A202G G207A A263G G499A A750G A827G A1438G A2706G G2831A T4117C A4769G G4820A T6413C C7028T G8206A 8281delC 8282delC 8283delC 8284delC 8285delC 8286delT 8287delC 8288delT 8289delA A8860G G11719A G13590A C14766T A15236G A15326G C15535T T16136C C16179N A16182N A16183N T16189C T16217C A16284G T16519C |
| NA18950 | A73G T146C A263G T310N T489C A750G A1438G A2706G T4254N A4715G A4769G C5100T G6179A C7028T C7196A G8584A C8684T A8701G A8860G T9540C T9758C A10398G C10400T T10873C A11560G G11719A A12017G C12705T T14470C C14766T T14783C G15043A G15301A A15326G A15487T C16134T A16170G C16184T C16223T T16298C G16319A |
| NA18951 | A73G A263G 310insC T489C 514delC 515delA A750G A1382C A1438G A2706G G3010A A4769G C4883T C5178A C6005T C7028T G8020A G8251A C8414T A8701G A8860G C8964T T9540C T9824A C10104T A10398G C10400T T10873C G11719A C12705T C14668T C14766T T14783C G15043A G15301A A15326G A15524G T15804C C16223T T16362C T16519C |
| NA18952 | A73G C150T T199C A263G T489C A750G A1438G A2706G G4048A C4071T G4113A A4164G A4769G A5351G G5460A C6455T T6680C C7028T G7269A T7684C G7853A A8701G A8860G T9540C T9824C T10345C A10398G C10400T T10873C G11719A C12405T C12705T T12811C C14766T T14783C G15043A G15301A A15326G G16129A T16189C C16223T T16297C T16298C |
| NA18953 | A73G A263G T489C G513A 514delC 515delA A750G T850N A1438G T2626C A2706G C2772T T4386C A4769G A4958G 5894insC C6455T C7028T G7852A A8701G A8860G T9540C T9824C A10398G C10400T T10873C T11017C A11084G G11719A C12705T G12771A T13768C G14364A C14766T T14783C G15043A G15301A A15326G T16209C C16223T T16324C |
| NA18955 | A73G T152C A263G A390G T489C G709A A750G A1438G A2706G T4688C A4769G A4833G T5108C C5601T C7028T G7600A A8701G A8860G C9168T A9377G T9540C G9575A A10398G C10400T T10873C G11719A G12192A C12705T C13383T A13563G G14569A C14766T T14783C G14861A G15043A G15301A A15326G A15562G C16223T A16227G C16234T C16278T T16362C |
| NA18956 | A73G A263G T489C A750G A1041G A1438G A2706G T3394C G4491A A4769G C7028T T7854C A8701G A8860G A9242G T9540C A10398G C10400T T10873C G11719A G11963A C12705T T14308C C14766T T14783C G15043A G15301A A15326G C16223T C16234T A16316G T16362C T16519C |
| NA18957 | A73G A263G T489C C542T A750G A1438G A2706G G3010A A4769G C4883T C5178A C7028T C8414T A8701G A8860G T9540C A10398G C10400T T10873C G11696A G11719A C12705T C14668T C14766T T14783C G15043A G15301A A15326G G16129A C16223T T16231C T16362C |
| NA18959 | A73G A263G T489C A750G A1438G G1709A A2706G G3010A A4769G C4883T C5178A C7028T C8414T A8701G A8860G T9540C A10398G C10400T T10873C G11696A G11719A C12705T T13656C C14668T C14766T T14783C G15043A T15139C G15301A A15326G C16184T C16223T T16311C T16362C A16525G |
| NA18960 | A73G C150T A263G 319insA A750G A1438G G1664A A2706G A4769G G4820A G5231A G5417A C7028T A8860G G11719A A12358G G12372A C12705T C14766T A15326G G16145A T16172C C16223T C16245T C16257A C16261T |
| NA18961 | A73G G103A A189G G203A T204C A263G 514delC 515delA G709A A750G A1438G G1598A A2623N T2626C A2706G A4769G G6962A C7028T 8281delC 8282delC 8283delC 8284delC 8285delC 8286delT 8287delC 8288delT 8289delA G8584A C8829T G8856A A8860G T9950C A10103G A10398G G11719A G12070A A12361G A14189G C14766T C15223T A15326G C15508T A15662G A15758G A15851G G15927A T16140C A16183C T16189C T16243C T16356C T16519C |
| NA18962 | A73G A263G T489C G709A A750G A1438G A2706G A4769G A4833G T5108C C5601T T5641C A6932G C7028T A8701G A8860G T8877C T9540C A10398G C10400T T10873C G11719A T11984C C12705T A13563G G14569A C14766T T14783C T14956C G15043A G15301A A15326G C16223T A16300G T16362C T16519C |
| NA18963 | A73G C150T A263G T489C G709A A750G A1438G A2706G A4769G A4793G A4833G T5108C C7028T A7646G C7867T T8200C A8701G A8860G T9540C A10398G C10400T T10873C C11549T G11719A G11914A C12705T G14569A C14766T T14783C G15043A G15301A G15323A A15326G G15497A A15860G C16223T T16325C T16362C T16519C |
| NA18964 | A73G T195C A235G A263G 514delC 515delA A663G A750G A1438G A1736G 2150insA A2706G T4248C G4655A A4769G A4824G C7028T A8563G C8794T A8860G C9488T C11536T C11647T G11719A C12705T C14766T A15326G T15519C C16187T C16223T C16290T C16295T G16319A T16519C |
| NA18965 | A73G A263G T489C 514delC 515delA A750G A1438G G1719A T2626C A2706G C2772T T4386C A4395G A4769G A4958G 5894insC 5894insC 5894insC 5894insC C6455T C7028T A8701G A8860G T9540C T9824C A10398G C10400T T10873C T11017C A11084G G11719A C12705T G12771A T13500C G14364A C14766T T14783C G15043A G15301A A15326G T16209C C16223T A16233G T16324C |
| NA18967 | A73G T146C C194T A263G T310C T489C 514delC 515delA A750G A1382C A1438G A2706G G3010N T4002C T4117C C4883T C5178A C7028T G8020A C8414T A8701G A8860N C8964T C9296T T9540C T9824A A10398G C10400T T10873C G11719A C12705T C13680T A14605G C14668T C14766T T14783C G15043A G15301A A15326G C16223T T16362C T16519C |
| NA18968 | A73G T199C T204C A263G T489C 514delC 515delA A750G A1382C A1438G A2706G G3010N A4769N C4883T C5178A C7028T G8020A G8251A C8414T C8595T A8701G A8901G C8964T T9540C T9824A C10104T A10352G A10398G C10400T T10873C G11719A C12705T T14287C C14668T C14766T T14783C G15043A G15301A A15326G A15524G T16189C C16223T T16362C |
| NA18969 | A73G A263G T489C A750G A1041G A1438G A2706G T3394C G4491A A4769G C7028T A8701G A8860N C8861N A9242G T9540C A10398G C10400T T10873C G11719A G11963A C12705T T14308C C14766T T14783C G15043A G15301A A15326G T15541C C16223T C16234T A16316G T16362C |
| NA18970 | A73G T152C 248delA A263G 514delC 515delA A750G A1438G A2706G C3970T T4705C A4732G A4769G C5049T G5147A T6392C G6962A C7028T A8860G G10310A T10609C C10976T G11719A G12406A C12633T C12882T G13928C G14476A C14766T A15326G A15954G G16129A A16182N A16183C T16189C C16232A T16249C T16304C T16311C C16344T T16519C |
| NA18971 | A73G A93G A210G A263G T310N 514delC 515delA A750G A1438G A2706G A3537G C7028T 8281delC 8282delC 8283delC 8284delC 8285delC 8286delT 8287delC 8288delT 8289delA G8584A T9950C G9962A A10398G G11149A C11151T G11719A A12234G C14149T C14158T C14766T A15235G A15326G G16129A T16140C C16187T T16189C C16266A T16519C |
| NA18972 | T60A A73G T146C C194T A263G T310N T489C 514delC 515delA A750G A1382C A1438G A2706G G3010A A4769N C4883T C5178A C7028T G8020A C8414T A8701G C8964T C9296T T9540C T9824A A10398G C10400T T10873C G11719A C12705T A14605G C14668T C14766T T14783C G15043A G15301A A15326G C16223T T16362C |
| NA18973 | A73G C150T A263G A750G T961C A1438G A2706G A4769G G5231A G5417A C7028T A8860G G11719A A12358G G12372A C12705T C14766T T14968C T15067C A15326G T16172C C16223T C16257A C16261T T16304C A16497G |
| NA18974 | A73G A263G T310C T482C A750G A1438G A2706G G5147N G5417A C5435T T6941C C7028T G7859A A10190G A10398G G11719A C12705T T14178C A14693G C14766T A14914G A15244G A15326G T16126C T16231C T16311C |
| NA18975 | A73C T89C A263G 308delC 309delC 310insC 514delC 515delA G709A A750G A1438G A2706G A4769G T5465C C7028T 8281delC 8282delC 8283delC 8284delC 8285delC 8286delT 8287delC 8288delT 8289delA A8860N G9123A T10238C G11719A C14766T C15292T A15326G C16148T 16182delA 16183delA T16189C T16217C C16261T T16519C |
| NA18976 | A73G C150T A263G A750G T961C A1438G A2706G G5231A G5417A C7028T G10685A G11719A A12358G G12372A C12705T A13857G C14766T T15067C A15326G T16172C C16223T C16250T C16257A C16261T T16519C |
| NA18977 | A73G T146C C194T A263G T489C 514delC 515delA A750G A1382C A1438G A2706G G3010A T4117C A4769G C4883T C5178A C7028T G8020A C8414T A8701G A8860G C8964T C9296T T9540C T9824A A10398G C10400T T10873C G11719A C12705T A14605G C14668T C14766T T14783C G15043A G15301A A15326G C16223T C16292T T16362C T16519C |
| NA18978 | G1N A2N T3N C4N A5N A73G A263G 311delC G316C T489C A750G A1438G A1945N C1946N C1947N C1948N G1949N T1950N A2706G G3010A A4769G C4883T C5178A C7028T C8414T A8701G G8856N G8857N G8858N C8859N A8860N C8861N A8862N G8863N T8864N G8865N A8866N T8867N T8868N A8869N T8870N A8871N T9540C A10398G C10400T T10873C C11215T G11719A C12705T C14668T C14766T T14783C G15043A G15301A A15326G A15874G G16129A C16223T T16362C |
| NA18979 | A73G A235G A263G 514delC 515delA A663G A750G A1438G A1736G 2150insA A2706G T4248C G4655A A4769G A4824G G5773A C7028T A8563G C8794T A8860G G10801A C11536T C11647T G11719A C12705T T12880C C14766T C14944T A15133G A15326G C16187T C16223T C16290T G16319A |
| NA18980 | A73G C150T C194T T195C A263G T489C 514delC 515delA A750G A1382C A1438G A2706G G3010A A4769G C4883T C5178A T6112C C7028T G8020A C8414T A8701G A8860G C8964T C9296T T9540C T9824A A10398G C10400T T10873C G11719A C12705T A14605G C14668T C14766T T14783C G15043A G15301A A15326G T16126C C16223T T16362C T16519C |
| NA18981 | A73G T152C A263G 310insC T489C A750G A1438G A2706G G3010N C3206T C4883T C5178A A5466G C7028T G7912A C8414T T8473C A8701G A8860N A9254N T9540C A10398G C10400T T10873C G11719A C12705T C14668T C14766T T14783C T14979C G15043A G15301A A15326G T16093C G16129A C16223T T16249C T16311C T16362C |
| NA18982 | A73G T146C A263G G709A A750G T1119C A1438G A2706G C3497T A4769G C7028T 8281delC 8282delC 8283delC 8284delC 8285delC 8286delT 8287delC 8288delT 8289delA A8860G G10310A G11719A A14133G C14766T A15326G G15346A A16183C T16189C T16217C T16311C T16519C |
| NA18983 | A73G T159C 190insA C194T T199C G207A A263G T310N T489C A750G A1438G A2706G C2766T G3010A G3391A A4769G C4883T C5178A C7028T C8414T A8701G A8860G T9540C G9755A A10398G C10400T T10873C G11719A C12705T C14668T C14766T T14783C G15043A G15301A A15326G C16245T T16362C |
| NA18984 | A73G T199C A202G G207A A263G T310N G499A 514delC 515delA A750G A827G A1438G T2685G A2706G G2831A T4117C A4769G G4820A T4967C G6023A T6413C C7028T G8206A 8281delC 8282delC 8283delC 8284delC 8285delC 8286delT 8287delC 8288delT 8289delA A8860G G10685A G11719A G13590A C14766T T15001C A15236G A15326G C15535T T16136C A16183C T16189C T16217C A16284G T16519C |
| NA18985 | A73G G103A T152C A189G T199C G203A T204C A263G T310N 514delC 515delA G709A A750G A1438G G1598A T2626C A2706G A4769G G6962A C7028T 8281delC 8282delC 8283delC 8284delC 8285delC 8286delT 8287delC 8288delT 8289delA G8584A C8829T G8856A A8860G T9950C T10003C A10103G A10398G G11719A C11881T A12361G G13477A C14766T C15223T A15326G C15508T A15662G A15851G G15927A T16136C T16140C A16170G 16183delA T16189C T16243C C16256T T16519C |
| NA18986 | A73G A235G A263G 514delC 515delA A663G A750G A1438G A1736G 2150insA A2706G T4248C G4655A A4769G A4824G C5396T G5773A C7028T A8563G C8794T A8860G G10801A C11536T C11647T G11719A C12705T T12880C C14766T C14944T A15326G C16187T C16223T C16290T G16319A |
| NA18987 | A73G A263G T310N T489C A750G A1148G A1438G A2706G G3010A A4769G C4883T C5178A T6620C C7028T C8414T A8701G A8730G T8762C A8860G T9540C A9667G A10398G C10400T T10873C G11719A C12088T G12651C C12705T C14668T C14766T T14783C G15043A G15301A A15326G G16244A T16311C T16362C |
| NA18988 | A73G C150T A263G T310N A750G A1438G G1664A A2706G A4769G G4820A G5231A G5417A C7028T A8860G G11719A A12358G G12372A C12705T T13641N C14766T A15326G G16145A T16172C C16223T C16245T C16257A C16261T |
| NA18989 | A73G G103A T199C T204C A263G T310N 514delC 515delA G709A A750G A1438G G1598A A2706G A4769G C7028T 8281delC 8282delC 8283delC 8284delC 8285delC 8286delT 8287delC 8288delT 8289delA G8584A A8784G C8829T A8860G T9950C A10398G C11146T G11719A G11914A G12192A A12361G T14470C C14766T C15223T A15326G C15508T A15662G A15851G G15927A A16183C T16189C C16223T T16243C A16318T G16319A T16362C T16519C |
| NA18990 | A73G C150T A263G T489C G709A A750G A1299G A1438G A2706G A4769G A4793G A4833G T5108C G5237N C7028T C7867T T8200C A8701G A8860G T9540C A10398G C10400T T10873C G11719A G11914A C12705T G13970N G14569A C14766T T14783C G15043A G15301A G15323A A15326G G15497A A15860G C16223T T16325C T16362C C16400T T16519C |
| NA18991 | A73G C150T A263G T310N A750G A1438G G1664A A2706G A4769G G4820A G5231A G5417A C7028T A8860G G11719A A12358G G12372A C12705T C14766T A15326G G16145A T16172C C16223T C16245T C16257A C16261T T16298C |
| NA18992 | A73G T152C A263G T489C A750G A1438G A2706G G3010A C3206T T3398C A4769N C4883T C5178A C7028T A7822G C8414T T8473C A8701G A8860G T9540C A10398G C10400T T10410C T10873C G11719A G11902A C12705T C14668T C14766T T14783C T14979C G15043A G15301A A15326G T16092C G16129A 16189delT C16223T T16362C T16519C |
| NA18993 | A73G A263G 286delA C431T T489C 514delC 515delA A750G A1438G A2706G G3010A A4769G T4859C C4883T C5178A C6410T C6689T C7028T G8020A C8414T A8701G A8860G T9540C T10084C C10181T A10398G C10400T T10873N G11719A A12172G C12705N T14635C C14668T C14766T T14783C G15043A G15301A A15326G T15440C A15951G C16223T C16287T G16319A T16362N A16399G |
| NA18994 | A73G A263G T489C A750G A1438G A2706G C2835T T3290C C3612T A4715G A4769G G6179A C7028T C7196A C7287T G8584A C8684T A8701G A8860G T9540C A10398G C10400T T10873C G11719A T12285C C12705T A14133G T14470C C14766T T14783C G15043A A15244G G15301A A15326G A15487T C16184T C16223T T16298C G16319A |
| NA18995 | A73G T152C A263G T489C A750G A1438G A2706G G3010A C3206T A4769N C4883T C5178A G5261A C7028T C8414T T8473C A8701G A8860G T9540C A10398G C10400T T10410C T10873C G11719A C12705T C14668T C14766T T14783C T14979C G15043A G15301A G15314A A15326G G16129A 16189delT C16223T T16362C T16519C |
| NA18997 | A73G G207A A235G A263G A663G A750G A1438G A1736G 2150insA A2706G T4248C G4655A A4769G A4824G C7028T A8563G C8794T A8860G G9755N C11536T C11647T G11719A C12705T A12909G C14766T A15326G T16172C C16187T C16223T C16290T G16319A |
| NA18998 | T10C A73G A263G T489C 514delC 515delA A750G A1382C A1438G A2706G G3010A A4769N C4883T C5178A C6005T C7028T G8020A G8251A C8414T A8701G A8860G C8964T T9540C T9824A C10104T A10398G C10400T T10873C G11719A A11884G C12705T A13105G C14668T C14766T T14783C G15043A G15301A A15326G A15524G C16223T T16362C |
| NA18999 | G1N A2N A73G A263G T489C 514delC 515delA A750G A1438G T2626C A2706G C2772T T4386C A4769G A4958G 5894insC C6455T C7028T A8701G A8860G A9299G T9540C T9824C A10398G C10400T T10873C T11017C A11084G G11719A T11722C C12705T G12771A G14364A C14766T T14783C G15043A G15301A A15326G T16209C C16223T T16324C |
| NA19000 | A73G A263G T489C 567insN A750G A1438G A2706G G3010A A4343G A4769G C4883T C5178A C7028T C8414T A8860G T9540C A10398G C10400T T10873C G11719A C12123T C12705T A13104G C14668T C14766T T14783C G15043A G15301A A15326G C15518T C16223T C16278T C16290T C16292T T16362C |
| NA19001 | A73G A263G T489C 514delC 515delA A750G A1438G T2626C A2706G C2772T G4048A T4386C A4769G A4958G 5894insC C6455T C7028T 8495delA 8496delT A8497T A8701G A8860G T9540C T9824C A10398G C10400T T10873C T11017C A11084G G11719A C12705T G12771A T14025C T14218C G14364A C14766T T14783C G15043A G15301A A15326G T16209C C16223T G16274A T16324C |
| NA19002 | A73G G103A C150T A263G T310N C456T T489C T681C A750G C1048T T1107C A1438G T2416C A2706G A3759G T4695C A4769G C4883T A5153G C5178A A5301G T6253C C7028T T7220C A8701G A8860G A9180G T9540C A10397G A10398G C10400T T10873C G11719A C12705T C14766T T14783C G15043A G15301A A15326G A15724G T16189C A16216G C16223T T16362C T16519C |
| NA19003 | A73G G260A A263G T489C G709A A750G A1438G A2706G C4505T A4769G A4833G T5108C C5601T A6737G C7028T G7600A A8701G A8860G T9165C A9377G T9540C G9575A A10398G C10400T T10873C G11719A T12311C C12705T A13563G T14200C C14281T G14569A C14766T T14783C G15043A G15301A A15326G A15758G T16189C C16223T C16278T T16362C |
| NA19004 | A73G A263G T489C 567insN G709A A750G A1438G A2706G T2885C G3010A A4343G A4769G C4883T C5178A C7028T C8414T A8860G T9540C A10398G C10400T T10873C G11719A C12705T A13104G C14668T C14766T T14783C G15043A G15301A A15326G C15518T C16223T C16278T T16362C |
| NA19005 | A73G C151T T152C 248delA A263G T489C A750G A1438G A2706G A4715G A4769G A6752G C7028T C7196A G8584A A8701G A8860G T9090C T9540C A10398G C10400T T10873C G11719A C12705T C14766T T14783C G15043A G15301A A15326G A15475G A15487T T15784C 15940delT C16185T C16223T C16260T T16298C A16302G |
| NA19007 | A73G C150T A263G T310N T482C T489C G709A A750G A1438G A2706G T4612C A4769G A4793G A4833G T5108C G5147A T6167G C7028T C7867T T8200C A8701G A8860G T9540C A10398G C10400T T10873C G11719A G11914A C12705T T14470C G14569A C14766T T14783C G15043A G15301A G15323A A15326G G15497A A15860G C16223T T16325C T16362C T16519C |
| NA19009 | A73G G103A T199C T204C A263G T310N 514delC 515delA G709A A750G 955insC A1438G G1598A A2706G A4769G A6929G C7028T 8281delC 8282delC 8283delC 8284delC 8285delC 8286delT 8287delC 8288delT 8289delA G8584A A8784G C8829T A8860G T9950C A10398G C11146T G11719A G11914A G12192A A12361G T14470C C14766T A14959G C15223T A15326G C15508T A15662G A15851G G15927A T16140C A16183C T16189C C16223T T16243C A16318T G16319A T16519C |
| NA19010 | A73G C194T A263G T310N T489C 514delC 515delA A750G A1382C A1438G A2617G A2706G G3010A A4769G C4883T C5178A C7028T G8020A C8414T A8701G A8860G C8964T C9296T T9540C T9824A A10398G C10400T T10873C G11719A C12705T A14605G C14668T C14766T T14783C G15043A G15301A A15326G C16223T T16362C T16519C |
| NA19012 | A73G T152C A235G A263G G499A 514delC 515delA A663G A750G A1438G A2706G T2857C T4248C A4769G A4824G C7028T C8794T A8860G A8962G T9148C C9711T G11719A C12705T G12771A A13140G C14766T A15326G C16223T C16290T G16319A T16519C |
| NA19054 | 42insC A73G C150T A263G T310N T489C 514delC 515delA A750G C752T T1107C C1310T A2706G A4769G C4883T C5178A A5301G C7028T C7888N A8701G A8860G A9180G T9540C A10397G A10398G C10400T T10873C G11719A T11944C A12026G C12398T C12705T A13278G C14766T T14783C G15043A G15301A A15326G T16092C A16164G A16182C A16183C T16189C C16223T C16266T T16356C T16362C |
| NA19055 | A73G C150T A263G T310N A750G A1438G A2706G A3729G A4769G G5231A G5417A A5498G C7028T A8860G G11719A A12358G G12372A C12705T C14766T A15326G G15883A T16172C T16189C T16209C C16223T C16257A C16261T |
| NA19056 | G1N A2N A73G A263G 310insC C431T T489C 514delC 515delA A750G A1438G A2706G G3010A A4769G T4859C C4883T C5178A C6410T C6689T C7028T G8020A C8414T A8701G A8860G T9540C T10084C C10181T A10398G C10400T T10873C G11719A A12172G C12705T C14668T C14766T T14783C G15043A G15301A A15326G T15440C A15951G C16287T G16319A T16362C A16399G |
| NA19057 | A73G A263G T489C A750G A1438G A2706G G3010A A4769G C4883T T5131C C5178A C7028T C8414T G8572A A8701G A8860G T9540C G9612A A10398G C10400T T10873C G11696A G11719A C12705T C14668T C14766T T14783C G15043A G15301A A15326G C16223T T16362C |
| NA19058 | A73G A263G T310N T489C G709A A750G A1438G A2706G G2831A A4769G A4833G T5108C C5601T C7028T G7600A A8701G A8860G A9377G T9540C G9575A A10398G C10400T T10873C G11719A C12705T A13563G T14200C G14569A C14766T T14783C G15043A G15301A A15326G T16189C C16223T A16227G C16278T T16362C T16519C |
| NA19059 | A73G T152C 248delA A263G 514delC 515delA A750G A1438G A2706G C3970T T4314C C4456T A4732G A4769G G5147A T6392C G6962A C7028T A8860G G10310A T10609C C10976T G11719A G12406A C12633T C12882T A13470G G13928C G14476A C14766T A15326G A15924G G16129A A16182C A16183C T16189C C16232A T16249C T16304C T16311C C16344T T16519C |
| NA19060 | T10C A73G C150T A263G T489C G709A A750G A1299G A1438G A2706G A4769G A4793G A4833G T5108C C7028T C7867T T8200C A8701G A8860G T9540C A10398G C10400T T10873C G11719A G11914A C12705T G14569A C14766T T14783C G15043A G15301A G15323A A15326G G15497A A15860G C16223T T16325C T16362C T16519C |
| NA19062 | A73G T146C T195C G207A A263G T489C G709A A750G A1438G A2706G A4769G A4833G T5108C C5601T C7028T G7600A T8063C A8701G A8860G G9266A A9377G T9540C G9575A A10018G A10398G C10400T T10873C G11719A C12705T A12753G A13563G T14200C G14569A C14766T T14783C G15043A G15301A A15326G A15758G C16223T A16227G C16278T T16362C |
| NA19063 | A73G T152C T195C A263G T489C T721A A750G A1438G A2706G G3010A T4254C A4769G C4883T C5178A C7028T C8414T A8701G A8860G T9540C A10398G C10400T T10873C G11719A C12705T A13681G C14668T C14766T T14783C G15043A G15301A A15326G C16223T T16362C |
| NA19064 | A73G T152C 248delA A263G T310N 514delC 515delA A750G A1438G A2706G C3970T A4732G A4769G C5049T G5147A T6392C G6962A C7028T A8860G G10310A T10609C C10976T G11719A G12406A C12633T C12882T G13928C G14476A C14766T A15326G A15954G G16129A A16182C A16183C T16189C C16232A T16249C T16304C T16311C C16344T T16519C |
| NA19065 | A73G T146C C150T T195C A214G A263G A750G T1119C A1438G G2056A A2706G C3497T G3531A A4769G A5441G C7028T 8281delC 8282delC 8283delC 8284delC 8285delC 8286delT 8287delC 8288delT 8289delA A8860G A10398G G11719A A13629G C14766T A15326G G15346A T15941C A16183C T16189C T16217C T16311C |
| NA19066 | A73G C194T T195C A263G T310N T489C 514delC 515delA A750G A1382C A1438G A2706G G3010A A4769G C4883T C5178A C7028T G8020A C8414T A8701G A8860G C8964T C9296T T9540C T9824A A10398G C10400T T10873C G11719A C12705T A14605G C14668T C14766T T14783C G15043A G15301A A15326G C16223T T16362C T16519C |
| NA19067 | A73G C150T A263G T489C G709A A750G A1438G A2706G A4769G A4793G A4833G T5108C C7028T C7867T T8200C A8563G A8701G A8860G T9540C A10398G C10400T T10873C G11719A G11914A C12705T G14569A C14766T T14783C G15043A G15301A G15323A A15326G G15497A A15860G C16223T T16325C T16362C T16519C |
| NA19068 | A73G A263G T310N T489C 514delC 515delA A750G G1007A A1438G A2706G G3010A T3336C T3644C G3915A A4769G C4883T T5048C C5178A C7028T C8414T A8701G A8860G T9540C A10398G C10400T T10873C A11092G G11719A C12705T G13708A C14668T C14766T T14783C G15043A G15301A A15326G C16174T C16223T A16241G T16362C |
| NA19070 | A73G C194T A263G T310N T489C 514delC 515delA A750G A1382C A1438G A2706G G3010A A4769G C4883T C5178A T6176C C7028T G8020A C8414T A8701G A8860G C8964T C9296T T9540C T9824A A10398G C10400T T10873C G11719A C12705T G13225A A14605G C14668T C14766T T14783C G15043A G15301A A15326G C16223T T16362C T16519C |
| NA19072 | A73G T152C A214G 248delA A263G T392C T489C G709A A750G T961C A1438G A2706G C3900T G4491A A4715G A4769G G4853A C5424T A6752G C7028T C7196A G8485A G8584A A8701G A8860G T9090C T9540C T10208C A10398G C10400T T10873C G11719A C12705T C14766T T14783C G15043A G15301A A15326G A15487T T15784C C16185T C16223T C16260T T16298C T16311C |
| NA19074 | A73G T146C C150T C151T T152C C182T T217C A263G T489C A750G T1107C A1438G A2706G C3546T A4200T T4216C A4769G C4883T C5178A A5301G C7028T A8701G A8860G T9540C A10397G A10398G C10400T T10873C G11719A C12705T A13105G C14766T T14783C A14927G G15043A G15301A A15326G T15622C T16093C 16188insC 16188insT T16189C T16325C T16362C G16390A T16519C |
| NA19075 | A73G C150T T199C A263G T310N T489C A750G A1438G A2706G G4048A C4071T A4164G A4769G A5351G G5460A C6455T T6680C C7028T T7684C G7853A A8701G A8860G T9540C T9824C T10345C A10398G C10400T T10873C G11719A C12405T C12705T T12811C C13011T C14766T T14783C G15043A G15301A A15326G G16129A T16189C C16223T T16297C T16298C |
| NA19076 | A73G T146C A263G T489C A750G A856G A1438G A2706G C2766T G3010A A4769G C4883T C5178A C7028T C7665T C8414T A8701G A8860G T9540C A10398G C10400T T10873C G11719A C12705T C14668T A14692G C14766T T14783C G15043A G15301A A15326G C16223T T16224C C16245T C16292T T16362C T16519C |
| NA19077 | A73G T199C A202G G207A A263G T310C G499A A750G A827G A1438G A2706G G2831A T4117C A4769G G4820A G6023A T6413C C7028T G7664A G8206A 8281delC 8282delC 8283delC 8284delC 8285delC 8286delT 8287delC 8288delT 8289delA A8860G G11719A G13590A C14766T A15236G A15326G C15535T T16136C A16183C T16189C T16217C A16284G T16324C T16519C |
| NA19078 | A73G C151T 248delA A263G 514delC 515delA C548T A750G A1438G A2706G G3915A C3970T C4086T A4769G A6254G T6392C G6962A C7028T A8860G G9053A G9548A C10211T G10310A T10609C G11719A G12406A C12882T G13759A G13928C C14766T G15314A A15326G T15629C G16129A A16162G T16172C T16304C T16519C |
| NA19079 | A73G A263G T310N A750G A1438G A2706G A4769G G5147A G5417A C7028T A7796G A8860G C10205T C10607T G11016A G11719A G12501A C12705T A13183G A13269G C14766T A14893G G14996A A15326G A16182C A16183C T16189C C16223T T16519C |
| NA19080 | A73G C150T A263G T489C G709A A750G A1438G A1625G A2706G A4769G A4793G A4833G T5108C C6164T T6167G C7028T C7867T T8200C G8485A A8701G A8860G G9380A T9540C A10398G C10400T T10873C G11719A G11914A C12705T G14569A A14575G C14766T T14783C G15043A G15301A G15323A A15326G G15497A A15860G C16223T T16325C T16362C T16519C |
| NA19081 | A73G T195C A235G C298T T310C T489C T504C 514delC 515delA A750G A1438G A2706G G3010A A4131G A4769G C4883T C5178A G5231A G5460A T6719C C7028T C8414T A8701G A8860G T9386C T9540C A10398G C10400T T10873C G11719A C12705T A13104G G13359A C14668T C14766T T14783C G15043A G15301A A15326G C16223T T16362C T16519C |
| NA19082 | A73G C150T A263G 514delC 515delA A750G T961C A1438G A2706G T4688A A4769G G5231A G5417A T5553G C7028T A8860G G11719A A12358G G12372A C12705T C14766T T15067C A15326G T16172C C16223T C16257A C16261T A16497G |
| NA19083 | A73G A263G T489C G709A A750G A1438G A2706G A4769G A4833G T5108C C5601T C7028T G7337A G7600A A8701G A8860G A9377G T9540C G9575A A10398G C10400T T10873C G11719A C12705T A13563G A13827G T14200C G14569A C14766T T14783C G15043A G15301A A15326G A16183C T16189C A16194N T16195C C16223T A16227G C16278T T16311C T16362C T16519C |
| NA19084 | A73G T152C 248delA A263G T310N T489C 514delC 515delA A750G A1438G A2706G A4715G A4769G A6752G C7028T C7196A G7337A G8584A A8701G A8860G T9090C T9540C T10208C A10398G C10400T T10873C G11719A C12705T C14766T T14783C G15043A G15301A A15326G A15487T T15784C C16185T C16223T C16260T T16298C |
| NA19085 | A73G T195C A263G T310N C431T T489C 514delC 515delA A750G A1438G A2706G G3010A A4769G T4859C C4883T C5178A C6410T C6689T C7028T G8020A C8414T A8701G A8730G A8860G T9540C T10084C C10181T A10398G C10400T T10873C G11719A A12172G C12705T C14668T C14766T T14783C G15043A G15301A A15326G T15440C A15951G C16223T C16287T G16319A T16362C A16399G |
| NA19086 | A73G T152C A263G T489C A750G A1438G A2246G A2706G G3010A A4769N C4883T C5178A C7028T A7673G G8020A C8414T T8450C A8701G A8860G T9540C C10181T A10398G C10400T T10873C G11719A C12705T A13827G G13928C A14091G C14668T C14766T T14783C A14927G G15043A G15217A G15301A A15326G T15440C A15805G A15951G C16223T G16319A T16362N |
| NA19087 | A73G G94A A214G A263G T334C T489C A750G A1438G A2706G G3010A G3316A A4769G C4883T G4959A C5178A T5964C C7028T C8414T A8701G A8860G T9495C C9536T T9540C C9620A A10398G C10400T T10873C C11215T G11719A C12705T T14470C C14668T C14766T T14783C G15043A G15301A A15326G A15924G C16223T T16362C |
| NA19088 | A73G T152C A263G T310N G709A A750G T1119C A1438G A2706G C3497T A4769G C7028T T7319C 8281delC 8282delC 8283delC 8284delC 8285delC 8286delT 8287delC 8288delT 8289delA A8860G G10310A A10978G G11719A A14133G A14452G C14766T A15326G G15346A A16182C A16183C T16189C T16217C T16311C T16519C |
| NA19089 | A73G 190insA C194N T199C G207A A263G T489C A750G A1438G A2706G C2766T G3010A G3391A A4769G C4883T C5178A C7028T C8414T A8701G A8860N C8861N G8863N T9084C T9540C G9755A A10398G C10400T T10873C G11719A C12705T C14668T C14766T T14783C G15043A G15301A A15326G C16245T T16362C |
| NA19546 | G1N A73G T152C A263G T489C A750G A1438G A2706G G3010A C3206T A4769N C4883T C5178A G5261A C7028T C8414T T8473C A8701G A8860G T9540C A10398G C10400T T10410C T10873C G11719A C12705T C14668T C14766T T14783C T14979C G15043A G15301A G15314A A15326G A15757G G15927A G16129A C16223T T16362C T16519C |
| NA19548 | A73G A263G T489C A750G A1438G T2626C A2706G C2772T T3552C T4386C A4769G A4958G 5894insC C6455T C7028T A8701G A8860G T9540C T9824C A10398G C10400T T10873C T11017C A11084G G11719A C12705T G12771A G14364A C14766T T14783C G15043A G15301A A15326G T16209C C16223T C16291T T16324C |
| NA19550 | G1N A73G T146C G185A A189G T196C A263G C308T 310delT G513A A750G A827G C1226N G1227N A1228N T1229N A1438G A2706G T4232C A4769N A4775N T6614C C7028T T7657C 8281delC 8282delC 8283delC 8284delC 8285delC 8286delT 8287delC 8288delT 8289delA G8584N C8585N A8586N G8587N T8588N A8589N C8590N T8591N G8592N A8593N A8860N G9921A A10750G T11353C G11719A C13464T C13705N C14109N T14110N C14766T A15326G C15535T G16129A G16153N A16170N A16171N T16172N C16173N C16174N A16175N C16176N A16177N T16178N C16179N A16180N A16181N A16182N A16183N C16184N C16185N C16186N C16187N C16188N T16189N C16190N C16191N C16192N C16193N A16194N T16217N C16223T A16247G C16320T T16519C |
| NA19551 | A73G A193G A263G 514delC 515delA G709A A750G A1438G A2706G A4769G T5201C T5465C C7028T 8281delC 8282delC 8283delC 8284delC 8285delC 8286delT 8287delC 8288delT 8289delA A8860N G9123A G11719A C14751T C14766T A15326G A16182N A16183C T16189C T16217C C16261T A16299G T16519C |
| NA19552 | T63N C64N G66N A73G 248delA A263G T310C 514delC 515delA A750G A1438G A2706G T2882C C3970T C4086T A4769G T6392C G6962A C7028T G9053A G9548A G10310A T10609C G11719A G12406A C12882T G13759A G13928C A14002G C14766T A15326G G16129A A16162G T16172C T16304C T16519C G16569N |
| NA19553 | A73G 248delA A263G 514delC 515delA A750G A1438G A2589G A2706G C3970T C4086T A4769G T6392C G6962A C7028T T8705C A8860G G9053A G9554A T9944C G10310A T10609C G11719A C11869T G12406A C12882T A13748G G13759A G13928C C14766T A15326G T15565C A16284G T16304C T16311C G16390A T16519C |
| NA19556 | G1N A2N T3N C4N A5N C6N A73G T152C A263G T310C 310insT C311N C312N T489C A750G A1438G A2706G G3010A C3206T A4769G C4883T C5178A T5201C G5261A C7028T C8414T T8473C A8701G A8860N T9540C A10398G C10400T T10410C T10873C G11719A C12705T C14668T C14766T T14783C T14979C G15043A G15301A G15314A A15326G G15803A G16129A C16223T T16362C T16519C |
| NA19558 | A73G C150T T152C T199C A263G T489C A750G A1438G A2706G G4048A C4071T A4164G A4769G A5351G G5460A G5979A C6455T T6680C C7028T T7684C G7853A A8701G A8860G T9540C T9824C T10345C A10398G C10400T T10873C G11719A C12405T C12705T T12811C C14766T T14783C G15043A G15301A A15326G G16129A C16169T T16189C C16223T T16297C T16298C G16566N A16567N T16568N G16569N |
| NA19560 | G1N A2N T3N C4N A5N C6N A73G A235G A263G 310insC 514delC 515delA A663G A750G A1438G A1736G 2150insA A2706G T4248C G4655A A4769G A4824G C7028T A8563G C8794T A8860N C11536T C11647T G11719A C12705T A12909G A14118G C14766T A15326G T15341C C16187T C16223T C16290T G16319A T16519C |
| NA19561 | G1N A2N A73G A235G A263G 514delC 515delA A663G A750G 955insN T961C A1438G G1709A A1736G A2706G T4248C A4769G A4824G C7028T A8563G C8794T A8860G A9545G C10637T C11536T G11719A C12705T C14766T C14983T A15326G T16126C C16223T C16290T G16319A T16519C G16569N |
| NA19563 | A73G T152C A263G T489C A750G A1041G A1438G A2706G T3394C G4491A A4769G C7028T A8701G A8860G A9242G T9540C A10398G C10400T T10873C G11719A G11963A C12705T T14308C C14766T T14783C G15043A G15301A A15326G C16223T C16234T A16316G T16362C |
| NA19564 | T63C C64T G66A A73G 248delA A263G 310delT 514delC 515delA A750G A1438G A2706G C3970T C4086T A4769G T6392C G6962A C7028T A8860G G9053A G9548A G10310A T10609C G11719A G12406A C12882T G13759A G13928C A14002G C14766T A15326G G16129A A16162G T16172C T16304C T16519C |
| NA19565 | A73G T152C A263G T489C A750G A1438G A2706G G3010A C3206T A4769G C4883T C5178A C7028T A7146G C8414T T8473C A8701G A8860G T9540C A10398G C10400T T10410C T10873C G11719A T11809C G11969A C12705T C14668T C14766T T14783C T14979C G15043A G15301A A15326G T15530C G16129A C16223T T16362C T16519C |
| NA19566 | G1N A73G A263G T489C C509N C510N C511N A512N G513N C514N A515N C516N A517N A750G A1438G T2626C A2706G C2772T T4386C A4769G A4958G T4978N A4979N A4980N A4981N C4989N 5894insC C6455T C7028N C7029N G7852A A8701G A8860G T9540C T9824C A10398G C10400T T10873C T11017C A11084G G11719A C12705T G12771A T13768C G14364A C14766T T14783C G15043A G15301A A15326G T16209C C16223T T16324C |
| NA19568 | G1N A2N A73G T146C A153G A263G T489C A750G A1438G A2706G G3010A 4315insT T4765N A4769N C4883T C5178A A6125G C7028T C8414T A8701G A8860N C8861N A8862N T9540C A10398G C10400T T10873C G11696A G11719A C12705T C14668T C14766T T14783C G15043A G15301A A15326G C16223T T16362C C16365T |
| NA19569 | G1N A2N T3N C4N A5N C6N A7N G8N G9N T10N C11N A73G A263G 310insC T489C A750G A1438G A2706G G3010A A4343G A4769G C4883T C5178A C7028T C8414T A8860N T9540C A10398G C10400T T10873C A11068G G11719A C12705T A13104G C14668T C14766T T14783C G15043A G15301A A15326G C15518T C16223T C16278T T16362C |
| NA19570 | A73G T152C 248delA A263G 514delC 515delA A750G A1438G A2706G C3970T A4732G A4769N G5147N T6392C G6962A C7028T G10310A T10609C C10976T G11719A G11887N G12406A C12633T C12882T G13928C G14476A C14629T C14766T A15326G G16129A A16182N A16183N C16184N C16185N T16189N C16232A T16249C T16304C T16311C C16344T T16519C |
| NA19572 | G1N A2N T3N C4N A73G T146C T152C A263G C308N T310N T489C A735G A750G A1438G A2706G G3010A C3206T A4769G C4883T C5178A C7028T C8414T T8473C A8701G A8860N T9540C A10398G C10400T T10410C T10873C G11719A C12705T C14668T C14766T T14783C T14979C G15043A G15301A A15326G G16129A C16223T T16362C T16519C |
| NA19573 | A73G A263G C296T 310insC T489C A750G A813G A1438G T1694N T2626C A2706G C2772T T4386C C4397N A4769G A4958G 5894insC C6455T C7028T A8701G A8860N T9540C T9824C A10398G C10400T T10873C T11017C A11084G G11719A C12705T G12771A G14364A C14766T T14783C G15043A G15301A A15326G T16209C C16223T C16291T T16324C |
| NA19574 | G1N A73G C150T A263G G709A A750G T1119C A1438G A2706G C3435T C3497T C3571T A4300G A4769G G5054C C7028T 8281delC 8282delC 8283delC 8284delC 8285delC 8286delT 8287delC 8288delT 8289delA A8860G T9128C G9575A T10493C G11440A G11719A C14766T A15326G G15346A T16136C T16140C T16189C T16217C T16249C G16274A C16291T A16335G T16519C |

Sites are according to rCRS. del means deletion and ins means insertion. Others represent substitutions.
